# A Novel Distributed State Estimation Algorithm with Consensus Strategy

**DOI:** 10.3390/s19092134

**Published:** 2019-05-08

**Authors:** Jun Liu, Yu Liu, Kai Dong, Ziran Ding, You He

**Affiliations:** 1 Research Institute of Information Fusion, Naval Aviation University, Yantai 264001, China; 18615042187@163.com (J.L.); 188dongkai@163.com (K.D.); DZR19931201@163.com (Z.D.); heyou_f@126.com (Y.H.); 2 School of Electronic and Information Engineering, Beihang University, Beijing 100191, China

**Keywords:** sensor networks, distributed state estimation, naive node, Kalman filter, maximum a posterior estimator, consensus filter

## Abstract

Owing to its high-fault tolerance and scalability, the consensus-based paradigm has attracted immense popularity for distributed state estimation. If a target is neither observed by a certain node nor by its neighbors, this node is naive about the target. Some existing algorithms have considered the presence of naive nodes, but it takes sufficient consensus iterations for these algorithms to achieve a satisfactory performance. In practical applications, because of constrained energy and communication resources, only a limited number of iterations are allowed and thus the performance of these algorithms will be deteriorated. By fusing the measurements as well as the prior estimates of each node and its neighbors, a local optimal estimate is obtained based on the proposed distributed local maximum a posterior (MAP) estimator. With some approximations of the cross-covariance matrices and a consensus protocol incorporated into the estimation framework, a novel distributed hybrid information weighted consensus filter (DHIWCF) is proposed. Then, theoretical analysis on the guaranteed stability of the proposed DHIWCF is performed. Finally, the effectiveness and superiority of the proposed DHIWCF is evaluated. Simulation results indicate that the proposed DHIWCF can achieve an acceptable estimation performance even with a single consensus iteration.

## 1. Introduction

Recently, distributed state estimation has been a hot topic in the field of target tracking in sensor networks [1,2,3,4,5,6,7,8,9,10,11,12,13,14,15,16,17,18,19]. As a traditional method, the centralized scheme needs to simultaneously process the local measurements from all sensors in the fusion center at each time instant [3,15]. This scheme guarantees the optimality of estimates, but a lot of communication and a powerful fusion center are required to maintain the operation, which may give rise to problems when the network size is increased or the communication resources are restricted. 

Unlike the centralized scheme, the distributed mechanism tries to recover the centralized performance via local communications between neighboring nodes. Specifically, each node in the network only exchanges information with its immediate neighbors to achieve a comparable performance to its centralized counterpart, which reduces communication cost and makes the network robust against possible failures of some nodes [8]. The consensus filter, which computes the average of interested values in a distributed manner, has attracted immense popularity for distributed state estimation [4,5,6,7,9,10,11,12,13,14,16,17,18,19,20,21,22]. Recently, in [23,24], the multiscale consensus scheme, in which the local estimated states achieve asymptotically prescribed ratios in terms of multiple scales, has been discussed and analyzed. The well-known Kalman consensus filter (KCF) [4,5,6,9,14] combines the local Kalman filter with the average consensus protocol together to update the posterior state. In the update stage, each node exploits the measurement innovations as well as the prior estimates from its inclusive neighbors (including the node itself and its immediate neighbors) to correct its prior estimate. However, the prior estimates from its immediate neighbors are assigned with same weights. This may ensure consensus on the estimates from different nodes after a period of time, but the estimation accuracy is not guaranteed. It is very likely that a target is neither observed by a certain node nor observed by any of its immediate neighbors. That is, there is no measurement in the inclusive neighborhood of the node, and it is naive about the target’s state. Similar to [16,22], such a node is referred as a naive node. Since a naive node contains less information about the target, it usually results in an inaccurate estimate. If a naive node is given an identical weight to the informed nodes, the final estimate will be severely contaminated, which may even cause the final estimates to diverge [9,13]. In addition, the cross covariances among different nodes are ignored in the derivation of KCF for computational and bandwidth requirements, and thus the covariance of each node is updated without regard to its neighbor’s prior covariance during the consensus step. Given no naive nodes in the network, KCF is able to provide satisfactory results. However, due to limited sensing abilities or constrained communication resources, a network often consists of some naive nodes. Especially in sparse sensor networks, this phenomenon is even more serious. In such a case, KCF would result in poor estimates [3]. To solve this problem, before updating the posterior estimate, the generalized Kalman consensus filter (GKCF) performs consensus on the prior information vectors and information matrices within the inclusive neighborhood of each node [4,16,19]. As is analyzed in [4], this procedure greatly improves the estimation performance in presence of naive nodes. GKCF updates current state based on consensus on prior estimates, but the current measurements are not considered for naive nodes. Each naive node only has access to measurements of the previous time instant existing in prior estimates. Therefore, there is a delay for naive nodes to access current measurements. On the contrary, consensus on measurements algorithm (CM) performs consensus on measurements [5,25,26,27], which can achieve the centralized performance with infinite consensus iterations. However, the stability is not guaranteed unless the number of consensus iterations is large enough [26]. Consensus on information algorithm (CI) performs consensus on both prior estimates and measurements [26,27,28], which can be viewed as a generalization of the covariance intersection fusion rule to multiple iterations [29]. CI guarantees stability for any number of consensus iterations, but its estimation confidence can be degraded as a conservative rule is adopted by assuming that the correlation between estimates from different nodes are completely unknown [26,28,30]. 

With more consensus iterations carried out, estimates from different nodes achieve a reasonable consensus. Therefore, each node has almost completely redundant or same prior information, and hence the prior estimation errors between nodes are highly correlated. In this situation, the algorithms such as KCF, GCKF, or CM, which do not take the cross-covariance into account, are sub-optimal [16,17]. Note that the redundant information only exists in the prior estimates, which come from the converged results in the previous time instant. Using this property, the information weighted consensus filter (ICF) [18,20,21] divides the prior information of each node by $N_{s}$ where $N_{s}$ is the total number of nodes in the network. If each node can interact with its neighbors for infinite times, ICF will achieve the optimal estimation performance as the centralized Kalman consensus filter (CKF). In addition, ICF performs better than KCF, GKCF, CI, and CM under the same consensus iterations, which has been validated in [16,22,26]. As is pointed out in [26,30,31], the correction step by multiplying $N_{s}$ may cause an overestimation of the measurement innovation for some nodes, which is often the case in sparse sensor networks. As a consequence, the estimates of some nodes may be too optimistic such that the estimation consistency will be lost, which should be avoided in recursive estimation. To address this problem, HCMCI algorithm combines the positive features of both CM and CI is proposed. It should be noted that HCMCI represents a family of different distributed algorithms dependent on the selection of scalar weights. Both CI and ICF are special cases of HCMCI. To preserve consistency of local filters as well as improve the estimation performance, the so-called normalization factor is introduced. If the network topology is fixed, the normalization factor can be computed offline to save bandwidth. In [32], a novel distributed set-theoretic information flooding (DSIF) protocol is proposed. The DSIF protocol benefits from avoiding the reuse of information and offering the highest converging efficiency for network consensus, but it suffers from growing requirements of node-storage, more communication iterations, and higher communication load.

However, it takes sufficient consensus iterations for the algorithms discussed above to achieve an expected estimation performance. In practical applications, only a limited number of consensus iterations is allowed, and thus the performance of the afore-mentioned algorithms is corrupted. In addition, the estimation performance of the afore-mentioned algorithms depends closely on the selection of consensus weights. Inappropriate consensus weights may cause the algorithms to diverge or require more iterations to achieve consensus on the local estimates [9]. It is a common way to set the weights as a constant value as discussed in [6], which is an intuitive choice to maintain the stability of the error dynamics. However, the constant value needs the knowledge of maximum degree across the entire sensor network. Even the maximum degree is available, it remains a problem how to determine a proper constant weight to achieve the best performance while preserving the property of consistency. In addition, the initial consensus terms determined in ICF require the knowledge of the total number of nodes in the network. The global parameters, such as the maximum degree or the total number of nodes, may vary over time when the communication topology is changed, some new nodes are joined, or some existing nodes fail to communicate with others. Without the accurate knowledge of these global parameters, each node would either overestimate or underestimate the state of interest.

To deal with the problems analyzed above, a novel distributed hybrid information weighted consensus filter (DHIWCF) is proposed in this paper. Firstly, different from the previous work [4,5,6,16,18,22], each node assigns consensus weights to its neighbors based on their local degrees, which is fully distributed with no requirement for any knowledge of the global parameters. Secondly, the prior estimate information and measurement information at current time instant within the inclusive neighborhood are, respectively, combined together to form the local generalized prior estimate equation and the local generalized measurement equation. Then, a distributed local MAP estimator is derived with some reasonable approximations of the error covariance matrices, which achieves higher accuracy than the approaches introduce in [4,5,6,11,16,18,19,25,26,27,28]. Finally, the average consensus protocol with the aforementioned consensus weights is incorporated into the estimation framework, and the proposed DHIWCF is obtained. In addition, the theoretical analysis on consistency of the local estimates, stability and convergence of the estimator is performed. The comparative experiments on three different target tracking scenarios validate the effectiveness and feasibility of the proposed DHIWCF. Even with a single consensus iteration, the DHIWCF is still able to achieve acceptable estimation performance. 

The remainder of this paper is organized as follows. Section 2 formulates the problem of distributed state estimation in sensor networks. The distributed local MAP estimator is derived in Section 3. Section 4 presents distributed hybrid information weighted consensus filter. The theoretical analysis on the consistency of estimates, stability and convergence of the estimator is provided in Section 5. The experimental results and analysis are considered in Section 6. The concluded remarks are given in Section 7.

Notation: ${\mathbb{R}}^{n}$ denotes the *n*-dimensional Euclidean space. ‖ ⋅ ‖ is the Euclidean norm in ${\mathbb{R}}^{n}$. For arbitrary matrix A, $A^{-1}$ and $A^{T}$ are respectively its inverse and transpose. A>0 means A is positive definite, and tr{A} is the shorthand for the trace of A. $\mathrm{diag}\left(B_{1},B_{2},\ldots ,B_{n}\right)$ denotes a block diagonal matrix with its main diagonal block being $B_{1},B_{2},\ldots ,B_{n}$. $I_{n}$ represents the n×n identity matrix. For a set C, |C| means the cardinality of C. E{⋅} is the expectation operator.

## 2. Problem Formulation

### 2.1. System Model

Consider a discrete time linear system with dynamics

(1) $$x_{k+1}=F_{k}x_{k}+w_{k}$$

where $x_{k}\in {\mathbb{R}}^{n_{x}}$ represents the state vector at time instant $k\in {\mathbb{Z}}^{+}$, where ${\mathbb{Z}}^{+}$ is the set of positive integers. $F_{k}$ is the state transition matrix. $w_{k}$ is the Gaussian process noise with zero-mean, covariance $Q_{k}$. 

The state of interest is observed by a sensor network consisting of $N_{s}$ nodes in the surveillance area. The measurement model of node i is

(2) $$z_{i,\;k}=H_{i,\;k}x_{k}+v_{i,k}$$

where $z_{i,\;k}\in {\mathbb{R}}^{n_{z}}$ is the measurement of node i at time instant k. $i=1,2,\ldots ,N_{s}$ denote the sensor labels. $H_{i,\;k}$ is the measurement matrix of node i. $v_{i,k}$ is the Gaussian measurement noise with zero mean, covariance $R_{i,k}$.

**Assumption** **1.** *The noise sequences ${\left\{w_{k}\right\}}_{k=\;0}^{\infty }$ and ${\left\{v_{i,k}\right\}}_{k=\;0}^{\infty }$ are mutually uncorrelated.*

**Assumption** **2** [16,18,22,26,27,28,30]**.** *If node i does not directly sense the target of interest at time instant k*, then*$R_{i,k}^{-1}=0$.*

**Remark** **1.** *Assumption 2 indicates that a node with no direct sensing ability is of infinite uncertainties about its local measurement, which guarantees consistency of the local measurements.*

### 2.2. Network Topology

The communication topology of the networked sensors can be represented by an undirected graph G=(S, ℰ). Here, $S=\left\{1,2,\ldots ,N_{S}\right\}$ is the set of sensor nodes, and ℰ⊆S×S is the available communication links in the network. A communication link means that any two neighboring nodes can exchange state or measurement information with each other. A connected network means that any node in the network may directly exchange information with at least one other node. The immediate neighbors of node i is denote by $N_{i}=\left\{j|\left(i,j\right)\in ℰ\right\}$. $d_{i}=\left|N_{i}\right|$ is degree of node i, which is the number of neighboring nodes linked to node i. The inclusive neighborhood which includes node i is represented by $J_{i}=\left\{i\right\}\cup N_{i}$. A more comprehensible way to describe the network topology is using adjacency matrix A, where element $a_{i,j}=1$ means that node i can exchange information with node j, and $a_{i,j}=0$ means that there is no direct communication link between node i and node j. The immediate neighbors of node i can be easily represented by $N_{i}=\left\{j|a_{i,j}=1\right\}$, and The degree of node i is $d_{i}=\sum_{j=1}^{N_{s}}a_{i,j}$.

**Definition** **1.** *If the target of interest is neither observed by node i nor observed by its neighbors $j\in N_{i}$, then node i is referred as a naive node. It should be noted that if node i is naive about the target of interest, $R_{j,k}^{-1}=0$ for $j\in J_{i}$ in view of Assumption 2.*

**Figure 1:**
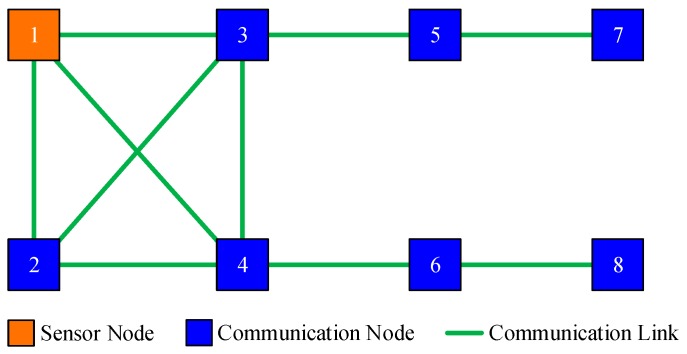
An illustrated sensor network with naive nodes.

For instance, there are 8 sensor nodes in the monitored area, and the communication topology is shown in Figure 1. Assume that only node 1 can directly observe the interested target, then nodes {2,3,4} can acquire state information from node 1 by local communication. However, there are no measurements within the inclusive neighborhood of nodes {5,6,7,8}, thus they are naive about the target’s state. 

From the perspective of adjacency matrix, the communication topology shown in Figure 1 can be simply represented by adjacency matrix A, where element $a_{i,j}=1$ means the available communication links in the illustrated network. It is easy to obtain the degree of each node from A. The neighbors of each nodes are also evident in A.

(3) $$A=\left[\begin{array}{llllllll} 0 & 1 & 1 & 1 & 0 & 0 & 0 & 0\\ 1 & 0 & 1 & 1 & 0 & 0 & 0 & 0\\ 1 & 1 & 0 & 1 & 1 & 0 & 0 & 0\\ 1 & 1 & 1 & 0 & 0 & 1 & 0 & 0\\ 0 & 0 & 1 & 0 & 0 & 0 & 1 & 0\\ 0 & 0 & 0 & 1 & 0 & 0 & 0 & 1\\ 0 & 0 & 0 & 0 & 1 & 0 & 0 & 0\\ 0 & 0 & 0 & 0 & 0 & 1 & 0 & 0 \end{array}\right]$$

### 2.3. Average Consensus

As an effective method to compute the mean value, the average consensus operates in a distributed fashion, which sheds light on the problem of distributed state estimation. Suppose the initial value of each node is $\alpha_{i}^{0}$. The goal is to compute the mean $\sum_{i=1}^{N_{s}}\alpha_{i}^{0}/N_{s}$ by local communications between neighboring nodes. At time instant k, node i sends its previous state $\alpha_{i}^{l-1}$ to its immediate neighbors $j\in N_{i}$, and in a similar way receives the previous state $\alpha_{j}^{l-1}$ from nodes $j\in N_{i}$. Then it updates its current state by the following fusion rule.

(4) $$\alpha_{i}^{l}=\alpha_{i}^{l-1}+\sum_{j\in N_{i}}\pi_{i,j}\left(\alpha_{j}^{l-1}-\alpha_{i}^{l-1}\right)$$

Here, $\pi_{i,j}$ is the consensus weight, which should satisfy certain conditions to ensure convergence to the mean of initial values [25,28,33]. A sufficient and necessary condition guaranteeing finite-time weighted average consensus has been provided in [34]. In the derivation of the proposed DHIWCF, the average consensus protocol is involved in the state update step, hence we only discuss the design of consensus weights ensuring average consensus on local estimates of all the nodes in the network.

If it is possible for the above protocol in (4) to iterate for infinite times, the estimated state of all nodes in the network will asymptotically converge to the average value, that is, $\underset{l\to \infty }{\lim}\alpha_{i}^{l}=\sum_{i=1}^{N_{s}}\alpha_{i}^{0}/N_{s}$. In the original KCF, the consensus rate is set to be a constant value $\varepsilon \in \left(0,\;1/d_{\max}\right)$, where $d_{\max}$ is the maximum node degree in the network [6].

**Remark** **2.** *A larger ε will accelerate the convergence of the protocol, but a ε equal to or more than $1/d_{\max}$ will render the protocol unstable [9,16]. The constant ε treats states from different nodes with the same weights, which may slow down the convergence rate of the whole system. The choice of ε depends on $d_{\max}$, which is not always available, especially in sparse sensor networks with time-varying communication topologies. In addition, there is no theoretical analysis on how to choose such a constant consensus weight.*

To avoid the requirements for global parameters and speed up convergence rate, the Metropolis weights determine consensus rate between neighboring nodes based on their local node degree. As is discussed in [30], Metropolis weights enable the protocol in (4) to achieve convergence faster. The definition of Metropolis weights is

(5) $$\pi_{ij}=\{\begin{array}{ll} {\left(1+\max\left\{d_{i},\;d_{j}\right\}\right)}^{-1}, & \mathrm{if}\;\left\{i,j\right\}\in ℰ\\ 1-\sum_{\left\{i,j\right\}\in ℰ}\pi_{ij}, & \mathrm{if}\;i=j\\ 0, & \mathrm{otherwise} \end{array}$$

**Remark** **3.** *The above definition in (5) indicates that a node with a larger degree will be assigned a smaller weight. All the consensus weights are computed only with the knowledge of local node degree, which is applicable to almost any kind of sensor networks. The interested reader is referred to [30] and the references therein for details. In this paper, the Metropolis weights are chosen for the proposed algorithm.*

The goal of the proposed DHIWCF is to achieve consensus on the local estimates of each node over the entire network by consensus iterations between neighboring nodes, and at the same time approach the estimation performance of CKF. If the network is fully connected, only a single iteration is enough to accomplish the estimation task. But in practical applications, a general network is often partially connected. To ensure the estimation accuracy and consensus on local estimates, it requires several iterations for the concerned information to spread throughout the entire network. However, due to constrained computation and communication resources, only a limited number of consensus iterations is available. It is urgent to design a more efficient distributed estimation scheme, which is able to achieve satisfactory estimation accuracy and consensus simultaneously with less consensus iterations.

## 3. Distributed Local MAP Estimation

This section starts with the centralized MAP estimator. Then we formulate the local generalized prior estimate equation based on prior estimates from the inclusive neighbors and the local generalized measurement equation based on the current measurements from the inclusive neighbors. By maximizing the local posterior probability, the local MAP estimator is derived. To implement the estimation steps in a distributed manner, approximation of the error cross covariance is required. Two special cases, where the prior errors from neighboring nodes are uncorrelated or completely identical, are considered here. The practical importance of such an approximation can be seen from the numerical examples in Section 6, which indicate that the proposed DHIWCF is effective even if the assumed cases are not fulfilled.

### 3.1. Global MAP Estimator 

Assume $z_{k}={\left[z_{1,\;k}^{T},z_{2,\;k}^{T},\ldots ,z_{N_{s},\;k}^{T}\right]}^{T}$ represents the collective measurements of the entire sensor network at time instant k. The stacked measurement matrix of all the nodes is denoted as $H_{k}={\left[H_{1,\;k}^{T},H_{2,\;k}^{T},\ldots ,H_{N_{s},\;k}^{T}\right]}^{T}$. The stacked measurement noise is $v_{k}={\left[v_{1,\;k}^{T},v_{2,\;k}^{T},\ldots ,v_{N_{s},\;k}^{T}\right]}^{T}$ with block diagonal covariance matrix $R_{k}=\mathrm{blkdiag}\left(R_{1,k},R_{2,k},\ldots ,R_{N_{s},k}\right)$. Then the global measurement model can be formulated as

(6) $$z_{k}=H_{k}x_{k}+v_{k}$$

Suppose the centralized prior estimate is ${\hat{x}}_{k|k-1}^{c}$. The corresponding prior estimation error is $e_{k|k-1}^{c}={\hat{x}}_{k|k-1}^{c}-x_{k}$ with covariance matrix $P_{k|k-1}^{c}=E\left\{e_{k|k-1}^{c}{\left(e_{k|k-1}^{c}\right)}^{T}\right\}$. Let ${\hat{x}}_{k|k}^{\mathrm{MAP}}$ be the maximum a posterior (MAP) estimate, we have

(7) $${\hat{x}}_{k|k}^{\mathrm{MAP}}=\underset{x_{k}}{\arg\max}p\left(x_{k}|Z_{k}\right)$$

where $p\left(z_{k}|Z_{k-1}\right)=\int p\left(z_{k}|x_{k}\right)p\left(x_{k}|Z_{k-1}\right)dx_{k}$ is a normalization constant. Since the process noise and measurement noise are both Gaussian, then the conditional PDF $p\left(z_{k}|x_{k}\right)$ and $p\left(x_{k}|Z_{k-1}\right)$ are also Gaussian. The explicit form of the prior PDF $p\left(x_{k}|Z_{k-1}\right)$ and the likelihood PDF $p\left(z_{k}|x_{k}\right)$ is formed as

(8) $$p\left(x_{k}|Z_{k}\right)=\frac{p\left(z_{k}|x_{k}\right)p\left(x_{k}|Z_{k-1}\right)}{p\left(z_{k}|Z_{k-1}\right)}$$

where $p\left(z_{k}|Z_{k-1}\right)=\int p\left(z_{k}|x_{k}\right)p\left(x_{k}|Z_{k-1}\right)dx_{k}$ is a normalization constant. Since the process noise and measurement noise are both Gaussian, then the conditional PDF $p\left(z_{k}|x_{k}\right)$ and $p\left(x_{k}|Z_{k-1}\right)$ are also Gaussian. The explicit form of the prior PDF $p\left(x_{k}|Z_{k-1}\right)$ and the likelihood PDF $p\left(z_{k}|x_{k}\right)$ is formed as

(9) $$p\left(x_{k}|Z_{k-1}\right)\propto \exp\left(-\frac{1}{2}\;{\left({\hat{x}}_{k|k-1}^{c}-x_{k}\right)}^{T}{\left(P_{k|k-1}^{c}\right)}^{-1}\left({\hat{x}}_{k|k-1}^{c}-x_{k}\right)]\right)$$

(10) $$p\left(z_{k}|x_{k}\right)\propto \exp\left(-\frac{1}{2}{\left(z_{k}-H_{k}x_{k}\right)}^{T}R_{k}^{-1}\left(z_{k}-H_{k}x_{k}\right)\right)$$

where ${\left\|x\right\|}_{A}^{2}=x^{T}Ax$. Based on Gaussian product in the numerator, the criterion in (7) can be reformulated by minimizing the following cost function.

(11) $${\hat{x}}_{k|k}^{\mathrm{MAP}}=\underset{x_{k}}{\arg\min}[{\left(z_{k}-H_{k}x_{k}\right)}^{T}R_{k}^{-1}\left(z_{k}-H_{k}x_{k}\right)+\;{\left({\hat{x}}_{k|k-1}^{c}-x_{k}\right)}^{T}{\left(P_{k|k-1}^{c}\right)}^{-1}\left({\hat{x}}_{k|k-1}^{c}-x_{k}\right)]$$

Here, the cost function in (11) is strictly convex on $x_{k}$ and hence the optimal ${\hat{x}}_{k|k}^{\mathrm{MAP}}$ is available.

(12) $${\hat{x}}_{k|k}^{\mathrm{MAP}}={\left({\left(P_{k|k-1}^{c}\right)}^{-1}+H_{k}^{T}R_{k}^{-1}H_{k}\right)}^{-1}\left({\left(P_{k|k-1}^{c}\right)}^{-1}{\hat{x}}_{k|k-1}^{c}+H_{k}^{T}R_{k}^{-1}z_{k}\right)$$

The corresponding posterior error covariance is

(13) $$P_{k|k}^{\mathrm{MAP}}={\left({\left(P_{k|k-1}^{c}\right)}^{-1}+H_{k}^{T}R_{k}^{-1}H_{k}\right)}^{-1}$$

The equivalent information form of the estimate in (12) and (13) can be rewritten as

(14) $${\hat{y}}_{k|k}^{\mathrm{MAP}}={\left(P_{k|k-1}^{c}\right)}^{-1}{\hat{x}}_{k|k-1}^{c}+H_{k}^{T}R_{k}^{-1}z_{k}$$

(15) $$Y_{k|k}^{\mathrm{MAP}}={\left(P_{k|k-1}^{c}\right)}^{-1}+H_{k}^{T}R_{k}^{-1}H_{k}$$

### 3.2. Local MAP Estimation

Assume that each node, for instance, node i, is able to receive its neighbor’s prior local estimate ${\hat{x}}_{j,k|k-1}$ and the corresponding covariance $P_{j,k|k-1}^{}$, as well as its neighbor’s local measurement $z_{j,k}$ and the corresponding noise covariance $R_{j,k}$ by local communication. The local generalized prior estimate, denoted by ${{\hat{x}}^{\prime }}_{i,k|k-1}^{}$, is defined as

(16) $${{\hat{x}}^{\prime }}_{i,k|k-1}^{}={\left[{\hat{x}}_{i,k|k-1}^{T},{\hat{x}}_{j_{1},k|k-1}^{T},\ldots ,{\hat{x}}_{j_{d_{i}},k|k-1}^{T}\right]}^{T}$$

where $j_{h}\;\in N_{i}\left(h=1,2,\ldots ,d_{i}\right)$ denotes the index of node i’s neighbors. Let $\eta_{i,k|k-1}^{}={\hat{x}}_{i,k|k-1}^{}-x_{k}^{}$ be the prior error of node i. The local collective prior error of node i with respect to its inclusive neighbors can be formulated as ${\eta^{\prime }}_{i,k|k-1}^{}={\left[\eta_{i,k|k-1}^{T},\eta_{j_{1},k|k-1}^{T},\ldots ,\eta_{j_{d_{i}},k|k-1}^{T}\right]}^{T}$. The local generalized prior estimate can be expressed by

(17) $${{\hat{x}}^{\prime }}_{i,k|k-1}^{}={ℋ}_{I}x_{k}^{}+{\eta^{\prime }}_{i,k|k-1}^{}$$

where ${ℋ}_{I}={\left[I_{p},I_{p},\ldots ,I_{p}\right]}^{T}$ is the matrix stacked by $d_{i}+1$ identity matrices. $x_{k}^{}$ is the true state at time instant k. The local collective prior error covariance of node i can be written as

(18) $${P^{\prime }}_{i,k|k-1}^{}=E\left\{{\eta^{\prime }}_{i,k|k-1}^{}{\left({\eta^{\prime }}_{i,k|k-1}^{}\right)}^{T}\right\}=\left[\begin{array}{llll} P_{i,k|k-1}^{} & P_{ij_{1},k|k-1}^{} & \cdots & P_{ij_{d_{i}},k|k-1}^{}\\ P_{j_{1}i,k|k-1}^{} & \ddots & & \vdots \\ \vdots & & \ddots & \vdots \\ P_{j_{d_{i}}i,k|k-1}^{} & \cdots & \cdots & P_{j_{d_{i}},k|k-1}^{} \end{array}\right]$$

Here, the block matrix ${P^{\prime }}_{i,k|k-1}^{}\in {\mathbb{R}}^{\left(1+d_{i}\right)n_{x}\times \left(1+d_{i}\right)n_{x}}$.

Similarly, the local generalized measurement of node i with regard to its inclusive neighbors can be formulated as

(19) $${z^{\prime }}_{i,\;k}={H^{\prime }}_{i,\;k}x_{k}+{v^{\prime }}_{i,k}$$

Here, ${z^{\prime }}_{i,\;k}={\left[z_{i,k}^{T},z_{j_{1},k}^{T},\ldots ,z_{j_{d_{i}},k}^{T}\right]}^{T}$ is the local generalized measurement. ${H^{\prime }}_{i,k}={\left[H_{i,\;k}^{T},H_{j_{1},\;k}^{T},\ldots ,H_{j_{d_{i}},\;k}^{T}\right]}^{T}$ is the local generalized measurement matrix. ${v^{\prime }}_{i,k}={\left[v_{i,\;k}^{T},v_{j_{1},\;k}^{T},\ldots ,v_{j_{d_{i}},\;k}^{T}\right]}^{T}$ denotes the local generalized measurement noise with covariance matrix ${R^{\prime }}_{i,k}=\mathrm{blkdiag}\left(R_{i,k},R_{j_{1},k},\ldots ,R_{j_{d_{i}},k}\right)$.

Combining (17) and (19) together, one has

(20) $$\left[\begin{array}{c} {{\hat{x}}^{\prime }}_{i,k|k-1}^{}\\ {z^{\prime }}_{i,\;k} \end{array}\right]=\left[\begin{array}{c} {ℋ}_{I}\\ {H^{\prime }}_{i,\;k} \end{array}\right]x_{k}+\left[\begin{array}{c} {\eta^{\prime }}_{i,k|k-1}^{}\\ {v^{\prime }}_{i,k} \end{array}\right]$$

where the error covariance

(21) $$E\left\{\left[\begin{array}{c} {\eta^{\prime }}_{i,k|k-1}^{}\\ {v^{\prime }}_{i,k} \end{array}\right]{\left[\begin{array}{c} {\eta^{\prime }}_{i,k|k-1}^{}\\ {v^{\prime }}_{i,k} \end{array}\right]}^{T}\right\}=\mathrm{blkdiag}\left({P^{\prime }}_{i,k|k-1}^{},\;{R^{\prime }}_{i,k}\right)$$

Here the operator blkdiag( ⋅ ) denotes the block diagonal matrix. 

According to the derivation of the global maximum a posterior estimator described in Section 3.1, the updated local information matrix can be computed by

(22) $$\begin{array}{ll} Y_{i,k|k}^{\mathrm{LMAP}} & ={\left[\begin{array}{c} {ℋ}_{I}\\ {H^{\prime }}_{i,\;k} \end{array}\right]}^{T}{\left[\begin{array}{cc} {P^{\prime }}_{i,k|k-1}^{} & 0\\ 0 & {R^{\prime }}_{i,k} \end{array}\right]}^{-1}\left[\begin{array}{c} {ℋ}_{I}\\ {H^{\prime }}_{i,\;k} \end{array}\right]\\ & ={ℋ}_{I}^{T}{\left({P^{\prime }}_{i,k|k-1}^{}\right)}^{-1}{ℋ}_{I}^{}+{\left({H^{\prime }}_{i,k}\right)}^{T}{\left({R^{\prime }}_{i,k}\right)}^{-1}{H^{\prime }}_{i,k}\\ & =\sum_{r=1}^{1+d_{i}}\sum_{s=1}^{1+d_{i}}{\left[{\left({P^{\prime }}_{i,k|k-1}^{}\right)}^{-1}\right]}_{r,s}+\sum_{j\in J_{i}}{\left(H_{j,\;k}\right)}^{T}{\left(R_{j,k}\right)}^{-1}H_{j,\;k} \end{array}$$

Similarly, the updated local information vector is

(23) $$\begin{array}{ll} {\hat{y}}_{i,k|k}^{\mathrm{LMAP}} & ={\left[\begin{array}{c} {ℋ}_{I}\\ {H^{\prime }}_{i,\;k} \end{array}\right]}^{T}{\left[\begin{array}{cc} {P^{\prime }}_{i,k|k-1}^{} & 0\\ 0 & {R^{\prime }}_{i,k} \end{array}\right]}^{-1}\left[\begin{array}{c} {{\hat{x}}^{\prime }}_{i,k|k-1}^{}\\ {z^{\prime }}_{i,\;k} \end{array}\right]\\ & ={ℋ}_{I}^{T}{\left({P^{\prime }}_{i,k|k-1}^{}\right)}^{-1}{{\hat{x}}^{\prime }}_{i,k|k-1}^{}+{\left({H^{\prime }}_{i,k}\right)}^{T}{\left({R^{\prime }}_{i,k}\right)}^{-1}{z^{\prime }}_{i,k}\\ & =\sum_{r=1}^{1+d_{i}}\sum_{s=1}^{1+d_{i}}{\left[{\left({P^{\prime }}_{i,k|k-1}^{}\right)}^{-1}\right]}_{r,s}{\left[{{\hat{x}}^{\prime }}_{i,k|k-1}^{}\right]}_{s}+\sum_{j\in J_{i}}{\left(H_{j,\;k}\right)}^{T}R_{j,k}^{-1}z_{j,\;k} \end{array}$$

Here, ${\left[{\left({P^{\prime }}_{i,k|k-1}^{}\right)}^{-1}\right]}_{r,s}$ denotes the (r,s)-th block matrix of ${\left({P^{\prime }}_{i,k|k-1}^{}\right)}^{-1}$. Similarly, ${\left[{{\hat{x}}^{\prime }}_{i,k|k-1}^{}\right]}_{s}$ denotes the s-th block vector of ${{\hat{x}}^{\prime }}_{i,k|k-1}^{}$.

### 3.3. Approximation of ${\left({P^{\prime }}_{i,k|k-1}^{}\right)}^{-1}$

It is shown in (22) and (23) that the key to acquire the local posterior estimate is to compute the inverse of the local collective prior error covariance, that is, ${\left({P^{\prime }}_{i,k|k-1}^{}\right)}^{-1}$. However, as is shown in (18), the computation of ${\left({P^{\prime }}_{i,k|k-1}^{}\right)}^{-1}$ requires the knowledge of cross-covariance between neighbors of node i. As is shown in [6], to compute the cross-covariance matrix $P_{ij_{h},k|k-1}^{}$, the information of the neighbors of node $j_{h}\;$ is also required. Therefore, it is not practical to directly compute ${\left({P^{\prime }}_{i,k|k-1}^{}\right)}^{-1}$ due to the fact that large amounts of communication among neighboring nodes are required, which may cause tremendous burden on computation and communication for the networked system. Although some work has been done in [35,36] to incorporate cross-covariance information into the estimation framework, no technique for computing the required terms are offered and predefined values are used instead [4]. 

Therefore, an approximation of ${P^{\prime }}_{i,k|k-1}^{}$ in a distributed manner is necessary. In the following derivation, two special cases are discussed. The first case is that the prior estimates from different nodes are completely uncorrelated with each other. This is true at the beginning of the estimation procedure when the prior information are initialized with random quantities. The second case is for converged priors, which is critical for the reason that with sufficient consensus iterations, the prior estimates from all nodes will converge to the identical value. 

#### 3.3.1. Case 1: Uncorrelated Priors

In this case, the prior errors from different nodes are assumed to be uncorrelated with each other, i.e., $E\left\{\eta_{i,k|k-1}^{}\eta_{j_{h},k|k-1}^{T}\right\}=0$. Hence, ${P^{\prime }}_{i,k|k-1}^{}$ in (18) turns into a block diagonal matrix ${P^{\prime }}_{i,k|k-1}^{}=\mathrm{blkdiag}\left(P_{i,k|k-1}^{},P_{j_{1},k|k-1}^{},\ldots ,P_{j_{d_{i}},k|k-1}^{}\right)$. The local posterior estimate in (22) and (23) can be approximated as

(24) $${\hat{y}}_{i,k|k}^{\mathrm{LMAP}}=\sum_{j\in J_{i}}Y_{j,k|k-1}^{}{\hat{x}}_{j,k|k-1}^{}+\sum_{j\in J_{i}}{\left(H_{j,\;k}\right)}^{T}R_{j,k}^{-1}z_{j,\;k}$$

(25) $$Y_{i,k|k}^{\mathrm{LMAP}}=\sum_{j\in J_{i}}Y_{j,k|k-1}^{}+\sum_{j\in J_{i}}{\left(H_{j,\;k}\right)}^{T}R_{j,k}^{-1}H_{j,\;k}$$

Note that after enough consensus iterations, the prior estimates of each node in the network asymptotically converges to the centralized result, i.e., $Y_{i,k|k-1}^{}=Y_{c,k|k-1}^{}$ and ${\hat{y}}_{i,k|k-1}^{}={\hat{y}}_{c,k|k-1}^{}$. In such a case, the local prior information matrix in (25) turns into $\sum_{j\in J_{i}}Y_{j,k|k-1}^{}=\left(1+d_{i}\right)Y_{c,k|k-1}^{}$. However, after convergence, the total amount of prior information in the network is $Y_{c,k|k-1}^{}$. That is, the local prior information matrix in the inclusive neighborhood is overestimated by a factor $\left(1+d_{i}\right)$. Therefore, the approximation of ${P^{\prime }}_{i,k|k-1}^{}$ should be modified by multiplying a factor $\left(1+d_{i}\right)$, which is ${P^{\prime }}_{i,k|k-1}^{}=\left(1+d_{i}\right)\mathrm{blkdiag}\left(P_{i,k|k-1}^{},P_{j_{1},k|k-1}^{},\ldots ,P_{j_{d_{i}},k|k-1}^{}\right)$ to avoid underestimation of the prior covariance. Hence, the results in (24) and (25) should be modified as

(26) $$Y_{i,k|k}^{}=\frac{1}{1+d_{i}}\sum_{j\in J_{i}}Y_{j,k|k-1}^{}+\sum_{j\in J_{i}}{\left(H_{j,\;k}\right)}^{T}R_{j,k}^{-1}H_{j,\;k}$$

(27) $${\hat{y}}_{i,k|k}^{}=\frac{1}{1+d_{i}}\sum_{j\in J_{i}}J_{j,k|k-1}^{}{\hat{x}}_{j,k|k-1}^{}+\sum_{j\in J_{i}}{\left(H_{j,\;k}\right)}^{T}R_{j,k}^{-1}z_{j,\;k}$$

#### 3.3.2. Case 2: Converged Priors

When the prior estimate of each node converges to the centralized result, one has

(28) $$\sum_{r=1}^{1+d_{i}}\sum_{s=1}^{1+d_{i}}{\left[{\left({P^{\prime }}_{i,k|k-1}^{}\right)}^{-1}\right]}_{r,s}=Y_{c,k|k-1}^{}=\frac{1}{1+d_{i}}\sum_{j\in J_{i}}Y_{c,k|k-1}^{}$$

Note that for converged priors, $Y_{j,k|k-1}^{}=Y_{c,k|k-1}^{},\;j\in J_{i}$. Substituting this fact into (28), there is

(29) $$\sum_{r=1}^{1+d_{i}}\sum_{s=1}^{1+d_{i}}{\left[{\left({P^{\prime }}_{i,k|k-1}^{}\right)}^{-1}\right]}_{r,s}=\frac{1}{1+d_{i}}\sum_{j\in J_{i}}Y_{j,k|k-1}^{}$$

Therefore, the estimated results in (22) and (23) can be transformed into the weighted summation of the prior information and current measurement innovations, which are the same forms as the results shown in (26) and (27).

**Remark** **4.** *It should be noted that the assumed cases are not always satisfied in realistic applications, but it is still of great significance for distributed filtering algorithms. The effectiveness and feasibility of such an approximation is evaluated by numerical examples in Section 6.*

## 4. Hybrid Information Weighted Consensus Filter

In Section 3, the prior estimate of each node is assumed to be known. Here, the prediction step is given.

(30) $${\hat{x}}_{i,k|k-1}^{}=F_{k-1}{\hat{x}}_{i,k-1|k-1}^{}$$

(31) $$Y_{i,k|k-1}^{}={\left(F_{k-1}Y_{i,\;k-1|k-1}^{-1}F_{k-1}^{T}+Q_{k-1}\right)}^{-1}$$

For simplicity, the prediction step in (31) can be rewritten as

(32) $$Y_{i,k|k-1}^{}=\Psi_{k-1}\left(Y_{i,\;k-1|k-1}^{}\right)$$

with

(33) $$\Psi_{k-1}\left(Y_{i,\;k-1|k-1}^{}\right)={\left(F_{k-1}Y_{i,\;k-1|k-1}^{-1}F_{k-1}^{T}+Q_{k-1}\right)}^{-1}$$

The corresponding prior information vector is

(34) $${\hat{y}}_{i,k|k-1}^{}=Y_{i,k|k-1}^{}{\hat{x}}_{i,k|k-1}^{}$$

With the above prediction steps and a weighted consensus protocol incorporated into the distributed local MAP estimator, a novel state estimation algorithm is obtained. Since the prior estimates and the measurement innovation are fused with different schemes, the proposed algorithm is referred as distributed hybrid information weighted consensus filter (DHIWCF). The recursive form of DHIWCF is detailed in Algorithm 1.

**Algorithm 1.** DHIWCF implemented by node i at time instant k.1. Obtain the local measurement $z_{i,\;k}$ with covariance matrix $R_{i,k}^{}$.2. Compute the measurement contribution vector and contribution matrix. (35)$$\{\begin{array}{l} u_{i}^{}=H_{i,k}^{T}R_{i,k}^{-1}z_{i,k}\\ U_{i}^{}=H_{i,k}^{T}R_{i,k}^{-1}H_{i,k}^{} \end{array}$$3. Broadcast state message $\left\{y_{i,k|k-1}^{},Y_{i,k|k-1}^{},u_{i}^{},U_{i}^{}\right\}$ to its neighboring nodes $j\in N_{i}$.4. Receive state message $\left\{y_{j,k|k-1}^{},Y_{j,k|k-1}^{},u_{j}^{},U_{j}^{}\right\}$ from its neighboring nodes $j\in N_{i}$.5. Compute the initial values. (36)$$\{\begin{array}{l} {\hat{y}}_{i,k|k}^{0}=\frac{1}{1+d_{i}}\sum_{j\in J_{i}}{\hat{y}}_{j,k|k-1}^{}+\sum_{j\in J_{i}}{\left(H_{j,\;k}\right)}^{T}R_{j,k}^{-1}z_{j,\;k}\\ Y_{i,k|k}^{0}=\frac{1}{1+d_{i}}\sum_{j\in J_{i}}Y_{j,k|k-1}^{}+\sum_{j\in J_{i}}{\left(H_{j,\;k}\right)}^{T}R_{j,k}^{-1}H_{j,\;k} \end{array}$$6. Perform consensus.  **for**
l=1:L
**do**
(1)Send ${\hat{y}}_{i,\;k|k-1}^{l-1}$ and $Y_{i,\;k|k-1}^{l-1}$ to its neighbors $j\in N_{i}$;(2)Receive ${\hat{y}}_{j,k|k-1}^{l-1}$ and $Y_{j,k|k-1}^{l-1}$ from its neighbors $j\in N_{i}$;(3)Update its consensus state.
(37)$$\{\begin{array}{l} {\hat{y}}_{i,\;k|k}^{l}={\hat{y}}_{i,\;k|k}^{l-1}+\sum_{j\in N_{i}}\pi_{i,j}\left({\hat{y}}_{j,\;k|k}^{l-1}-{\hat{y}}_{i,\;k|k}^{l-1}\right)\\ Y_{i,\;k|k}^{l}=Y_{i,\;k|k}^{l-1}+\sum_{j\in N_{i}}\pi_{i,j}\left(Y_{j,\;k|k}^{l-1}-Y_{i,\;k|k}^{l-1}\right) \end{array}$$  **end for**7. Compute the posterior estimate.${\hat{x}}_{i,k|k}^{}={\left(Y_{i,\;k|k}^{L}\right)}^{-1}{\hat{y}}_{i,\;k|k}^{L}$, $Y_{i,k|k}^{}=Y_{i,k|k}^{L}$, ${\hat{y}}_{i,\;k|k}^{}={\hat{y}}_{i,\;k|k}^{L}$8. Prediction at time instant k+1${\hat{x}}_{i,k+1|k}^{}=F_{k}{\hat{x}}_{i,k|k}^{}$, $Y_{i,k+1|k}^{}=\Psi_{k}\left(Y_{i,\;k|k}^{}\right)$, ${\hat{y}}_{i,k+1|k}^{}=Y_{i,k+1|k}^{}{\hat{x}}_{i,k+1|k}^{}$

## 5. Performance Analysis

### 5.1. Consistency of Estimates

One of the most fundamental but important properties of a recursive filtering algorithm is that the estimated error statistics should be consistent with the true estimation errors. The approximated error covariance of an inconsistent filtering algorithm is too small or optimistic, which does not really indicate the uncertainty of the estimate and may result in divergence since subsequent measurements in this case are prone to be neglected [28].

**Definition** **2** [28,30,37,38]**.** *Consider a random vector x. Let $\hat{x}$ and P be, respectively, the estimate of x and the estimate of the corresponding error covariance. Then the pair $\left(\hat{x},P\right)$ is said to be consistent if*

(38) $$E\left\{\left({\hat{x}}_{}-x_{}\right){\left({\hat{x}}_{}-x_{}\right)}^{T}\right\}\leq P$$

It is shown in (38) that consistency requires that the true error covariance should be upper bounded (in the positive definite sense) by the approximated error covariance P. In the distributed estimation paradigm, due to the unaware reuse of the redundant data in the consensus iteration and the possible correlation between measurements from different nodes, the filter may suffer from inconsistency and divergence. In such a case, preservation of consistency is even much more important. 

For convenience, consider the information pair $\left(\hat{y},Y\right)$, where $\hat{y}=P^{-1}\hat{x}$ and $Y=P^{-1}\hat{x}$. The consistency defined by (38) can be rewritten as

(39) $$Y\leq {\left(E\left\{\left(Y^{-1}\hat{y}-x_{}\right){\left(Y^{-1}\hat{y}-x_{}\right)}^{T}\right\}\right)}^{-1}$$

**Assumption** **3.** *The initialized estimate of each node, represented by $({\hat{x}}_{i,0|0}^{},P_{i,0|0}^{}),i\in S$, is consistent. Equivalently, inequality $P_{i,0|0}^{}\geq E\left\{\left({\hat{x}}_{i,0|0}^{}-x_{0}\right){\left({\hat{x}}_{i,0|0}^{}-x_{0}\right)}^{T}\right\}$ holds for i∈S.*

**Remark** **5.** *In general, Assumption 3 can be easily satisfied. The initial information on the state vector can be acquired in an off-line fashion before the fusion process. In the worst case where no prior information is available, each node can simply set the initialized information matrix as $P_{i,0|0}^{-1}=0$, which implies infinite estimate uncertainty in each node at the beginning so that Assumption 3 is fulfilled.*

**Assumption** **4.** *The system matrix $F_{k}$ is invertible.*

**Lemma** **1** [28]**.** *Under Assumption 4, if two positive semidefinite matrices $Y_{1}$ and $Y_{2}$ satisfy $Y_{1}\leq Y_{2}$, then $0\leq \Psi_{k}\left(Y_{1}\right)\leq \Psi_{k}\left(Y_{2}\right)$. In other words, the function $\Psi_{k}\left(\;\cdot \;\right)$ is monotonically nondecreasing for any k≥0.*

**Theorem** **1.** *Let Assumptions 1, 2, and 3 hold. Then, for each time instant k and each node i∈S, the information pair $\left({\hat{y}}_{i,k|k},Y_{i,k|k}\right)$ of the DHIWCF is consistent in that*

(40) $$Y_{i,k|k}^{}\leq {\left(E\left\{\left({\hat{x}}_{i,k|k}^{}-x_{k}\right){\left({\hat{x}}_{i,k|k}^{}-x_{k}\right)}^{T}\right\}\right)}^{-1}$$

*with*${\hat{x}}_{i,k|k}^{}=Y_{i,k|k}^{-1}{\hat{y}}_{i,\;k|k}^{}$.

**Proof.**  An inductive method is utilized here to prove this theorem. It is supposed that, at time instant k−1

(41) $$Y_{i,k-1|k-1}^{}\leq {\left(E\left\{\left({\hat{x}}_{i,k-1|k-1}^{}-x_{k-1}\right){\left({\hat{x}}_{i,k-1|k-1}^{}-x_{k-1}\right)}^{T}\right\}\right)}^{-1}$$

for any i∈S. For brevity, the predicted information matrix in (31) can be rewritten as

(42) $$Y_{i,k|k-1}^{}=\Psi \left(Y_{i,\;k-1|k-1}^{}\right)$$

On the basis of Lemma 1, it is immediate to see

(43) $$\begin{array}{ll} Y_{i,k|k-1}^{} & =\Psi_{k-1}\left(Y_{i,\;k-1|k-1}^{}\right)\\ & \leq \Psi_{k-1}\left({\left(E\left\{\left({\hat{x}}_{i,k-1|k-1}^{}-x_{k-1}\right){\left({\hat{x}}_{i,k-1|k-1}^{}-x_{k-1}\right)}^{T}\right\}\right)}^{-1}\right)={\left(E\left\{\left({\hat{x}}_{i,k|k-1}^{}-x_{k}\right){\left({\hat{x}}_{i,k|k-1}^{}-x_{k}\right)}^{T}\right\}\right)}^{-1} \end{array}$$

According to (26) and (27), the local estimation error is

(44) $$\begin{array}{ll} {\hat{x}}_{i,k|k}^{0}-x_{k} & ={\left(Y_{i,k|k}^{0}\right)}^{-1}{\hat{y}}_{i,k|k}^{0}-{\left(Y_{i,k|k}^{0}\right)}^{-1}Y_{i,k|k}^{0}x_{k}\\ & ={\left(Y_{i,k|k}^{0}\right)}^{-1}\left[\frac{1}{1+d_{i}}\sum_{j\in J_{i}}Y_{j,k|k-1}^{}\left({\hat{x}}_{j,k|k-1}^{}-x_{k}\right)+\sum_{j\in J_{i}}{\left(H_{j,\;k}\right)}^{T}R_{j,k}^{-1}\left(z_{j,\;k}-H_{j,\;k}x_{k}\right)\right]\\ & ={\left(Y_{i,k|k}^{0}\right)}^{-1}\left[\frac{1}{1+d_{i}}\sum_{j\in J_{i}}Y_{j,k|k-1}^{}\left({\hat{x}}_{j,k|k-1}^{}-x_{k}\right)+\sum_{j\in J_{i}}{\left(H_{j,\;k}\right)}^{T}R_{j,k}^{-1}v_{j,\;k}\right] \end{array}$$

Then,

(45) $${\left(E\left\{\left({\hat{x}}_{i,k|k}^{0}-x_{k}\right){\left({\hat{x}}_{i,k|k}^{0}-x_{k}\right)}^{T}\right\}\right)}^{-1}={\left({\left(Y_{i,k|k}^{0}\right)}^{-1}\left(E\left\{\Delta_{k,i}\right\}+\sum_{j\in J_{i}}{\left(H_{j,\;k}\right)}^{T}R_{j,k}^{-1}H_{j,\;k}\right){\left(Y_{i,k|k}^{0}\right)}^{-1}\right)}^{-1}$$

where

(46) $$\Delta_{k,i}=\left(\sum_{j\in J_{i}}\frac{1}{1+d_{i}}Y_{j,k|k-1}^{}\left({\hat{x}}_{j,k|k-1}^{}-x_{k}\right)\right){\left(\sum_{j\in J_{i}}\frac{1}{1+d_{i}}Y_{j,k|k-1}^{}\left({\hat{x}}_{j,k|k-1}^{}-x_{k}\right)\right)}^{T}$$

According to the consistency property of covariance intersection [29,38], it holds that

(47) $$E\left\{\Delta_{k,i}\right\}\leq \sum_{j\in J_{i}}\frac{1}{1+d_{i}}Y_{j,k|k-1}^{}E\left\{\left({\hat{x}}_{j,k|k-1}^{}-x_{k}\right){\left({\hat{x}}_{j,k|k-1}^{}-x_{k}\right)}^{T}\right\}Y_{j,k|k-1}^{}\leq \frac{1}{1+d_{i}}\sum_{j\in J_{i}}Y_{j,k|k-1}^{}$$

Then, exploiting (47) and (43) in (45), the following result is obtained.

(48) $${\left(E\left\{\left({\hat{x}}_{i,k|k}^{0}-x_{k}\right){\left({\hat{x}}_{i,k|k}^{0}-x_{k}\right)}^{T}\right\}\right)}^{-1}\geq {\left({\left(Y_{i,k|k}^{0}\right)}^{-1}\left(\frac{1}{1+d_{i}}\sum_{j\in J_{i}}Y_{j,k|k-1}^{}+\sum_{j\in J_{i}}{\left(H_{j,\;k}\right)}^{T}R_{j,k}^{-1}H_{j,\;k}\right){\left(Y_{i,k|k}^{0}\right)}^{-1}\right)}^{-1}\geq Y_{i,k|k}^{0}$$

Since the information pair $\left({\hat{x}}_{i,k|k}^{l+1},Y_{i,k|k}^{l+1}\right)$ is computed based on the previous information pair $\left({\hat{x}}_{i,k|k}^{l},Y_{i,k|k}^{l}\right)$ by (3), and the covariance intersection involved in (3) preserves the consistency of estimates [29,37,38,39], it can be concluded that ${\left(E\left\{\left({\hat{x}}_{i,k|k}^{l}-x_{k}\right){\left({\hat{x}}_{i,k|k}^{l}-x_{k}\right)}^{T}\right\}\right)}^{-1}\geq Y_{i,k|k}^{l}$ indicates ${\left(E\left\{\left({\hat{x}}_{i,k|k}^{l+1}-x_{k}\right){\left({\hat{x}}_{i,k|k}^{l+1}-x_{k}\right)}^{T}\right\}\right)}^{-1}\geq Y_{i,k|k}^{l+1}$ for any l=1,…,L. In other words, if the estimate obtained with *l* consensus iterations is consistent, the estimate obtained with *l* + 1 consensus iterations is also consistent. Therefore, it is straightforward to conclude that (40) holds with ${\hat{x}}_{i,k|k}={\hat{x}}_{i,k|k}^{L}$ and $Y_{i,k|k}=Y_{i,k|k}^{L}$. The proof is concluded since the initial estimate ${\hat{x}}_{i,0|0},\forall i\in S$ is consistent. □

### 5.2. Boundedness of Error Covariances

According to the consistency of the proposed DHIWCF in Theorem 1, it is sufficient to prove that $Y_{i,k|k}^{}$ is lower bounded by a certain positive matrix (or equivalently, to prove $P_{i,k|k}^{}=Y_{i,k|k}^{-1}$ is upper bounded by some constant matrix) for the proof of the boundedness of the error covariance $E\left\{\left({\hat{x}}_{i,k|k}^{}-x_{k}\right){\left({\hat{x}}_{i,k|k}^{}-x_{k}\right)}^{T}\right\}$. To derive the bounds for the information matrix $Y_{i,k|k}^{}$, The following assumptions are required.

**Assumption** **5.** *The system is collectively observable. That is, the pair $\left(F_{k},H_{k}\right)$ is observable where $H_{k}=\mathrm{col}\left(H_{i,\;k},\;i\in S\right)$.*

Let Π be the consensus matrix, whose elements are the consensus weights $\pi_{i,j}^{}$ for any i,j∈S. Further, let $\pi_{i,j}^{L}$ be the (i,j)-th element of $\Pi^{L}$, which is the L-th power of Π.

**Assumption** **6.** *The consensus matrix Π is row stochastic and primitive.*

**Assumption** **7.** *There exist real numbers $\underline{f},\bar{f},\underline{h},\bar{h}\neq 0$ and positive real numbers $\bar{p}>\underline{p}>0$, $\bar{q}>\underline{q}>0$, such that the following bounds are fulfilled for each k≥0, i∈S.*

(49) $$\{\begin{array}{l} {\underline{f}}^{2}I_{n}\leq F_{k}F_{k}^{T}\leq {\bar{f}}^{2}I_{n},\;{\underline{h}}^{2}I_{m}\leq H_{i,k}{\left(H_{i,k}\right)}^{T}\leq {\bar{h}}^{2}I_{m}\\ \underline{q}I_{n}\leq Q_{k}\leq \bar{q}I_{n},\;\underline{r}I_{m}\leq R_{i,k}\leq \bar{r}I_{m} \end{array}$$

**Lemma** **2** [28]**.** *Under Assumptions 4 and 5, and the proposed DHIWCF algorithm, if there exists a positive semidefinite matrix $\overset{⌣}{Y}$ such that $Y_{i,k|k}^{}\leq \overset{⌣}{Y},\;\forall k\geq 0,\;i\in S$, then there always exists a strictly positive constant 0<α<1 such that*

(50) $$Y_{i,k+1|k}^{}\geq \alpha {\left(F_{k}\right)}^{-T}Y_{i,\;k|k}^{}F_{k}^{-1}$$

By virtue of Lemma 2, Theorem 2 which depicts the boundedness of error covariances is presented below.

**Theorem** **2.** *Let Assumptions 4–7 hold, there exist positive definite matrices $\underline{\Omega }$ and $\bar{\Omega }$ such that*

(51) $$0<\underline{\Omega }\leq Y_{i,\;k|k}^{}\leq \bar{\Omega },\;\forall k\geq 0,\;i\in S$$

*where $Y_{i,\;k|k}^{}$ is the information matrix given by the proposed DHIWCF.*

**Proof.**  For simplicity, the proof is concluded for the case L=1. The generalization for L>1 can be directly derived in a similar way. According to the proposed DHIWCF, the information matrix for node i at time instant k can be written as

(52) $$Y_{i,k|k}^{}=\sum_{j\in S}\sum_{r\in J_{j}}\pi_{i,j}^{}{\left(1+d_{j}\right)}^{-1}Y_{r,k|k-1}^{}+\sum_{j\in S}\sum_{r\in J_{j}}\pi_{i,j}^{}{\left(H_{r,\;k}\right)}^{T}R_{r,k}^{-1}H_{r,\;k}$$

In view of Assumption 6, 7 and fact that $Y_{r,k|k-1}^{}\leq Q_{k-1}^{-1}$ by (31), one can get

(53) $$\begin{array}{ll} Y_{i,k|k}^{} & \leq Q_{k-1}^{-1}+\sum_{j\in S}\sum_{r\in J_{j}}\pi_{i,j}^{}{\left(H_{r,\;k}\right)}^{T}R_{r,k}^{-1}H_{r,\;k}\\ & \leq \left(\frac{1}{\underline{q}}+\frac{\left(1+d_{\max}\right){\bar{h}}^{2}}{\underline{r}}\right)I_{n_{x}}≜\bar{\Omega } \end{array}$$

Hence, the upper bound is achieved. Next a lower bound will be guaranteed under Assumption 5.

According to Lemma 2 and Assumption 7 and (31), (53), it follows from (52) that

(54) $$Y_{i,k|k}^{}\geq \alpha \sum_{j\in S}\sum_{r\in J_{j}}\pi_{i,j}^{}{\left(1+d_{j}\right)}^{-1}{\left(F_{k-1}\right)}^{-T}Y_{r,\;k-1|k-1}^{}F_{k-1}^{-1}+\sum_{j\in S}\sum_{r\in J_{j}}\pi_{i,j}^{}{\left(H_{r,\;k}\right)}^{T}R_{r,k}^{-1}H_{r,\;k}$$

where α is a positive scalar with 0<α<1. By recursively exploiting (52) and (54) for a certain number (denoted by $\bar{k}$) of times, there is

(55) $$\begin{array}{ll} Y_{i,k|k}^{} & \geq \alpha^{\bar{k}}\sum_{j\in S}\sum_{r\in S}\pi_{i,j}^{\bar{k}}\Xi_{j,\;r}^{\bar{k}}{\left(\underset{\mathrm{length}=\bar{k}}{\underbrace{F_{k-1}F_{k-2}\ldots F_{k-\bar{k}}}}\right)}^{-T}Y_{i,\;k-1|k-1}^{}{\left(F_{k-1}F_{k-2}\ldots F_{k-\bar{k}}\right)}^{-1}\\ & +\sum_{\tau =1}^{\bar{k}}\alpha^{\tau -1}{\left(\underset{\mathrm{length}=\;\tau -1}{\underbrace{F_{k-1}F_{k-2}\ldots F_{k-\tau +1}}}\right)}^{-T}\left(\sum_{j\in S}\sum_{r\in S}\pi_{i,j}^{\tau }\Xi_{j,\;r}^{\tau -1}{\left(H_{r,\;k-\tau +1}\right)}^{T}R_{r,k-\tau +1}^{-1}H_{r,\;k-\tau +1}\right){\left(F_{k-1}F_{k-2}\ldots F_{k-\tau +1}\right)}^{-1} \end{array}$$

where $\pi_{i,j}^{\tau }$ is the (i,j)-th element of $\Pi^{\tau }$. Ξ is a matrix with elements

(56) $$\Xi_{i,j}=\{\begin{array}{l} 1/\left(1+d_{i}\right),\;\mathrm{if}\;j\in J_{i}\\ 0,\;\mathrm{otherwise} \end{array}$$

Note that the matrix Ξ is constructed based on the network topology and is naturally stochastic. According to [40,41], as long as the undirected network is connected, similar to the definition of Π, Ξ is primitive. Therefore, there exist strictly positive integers m and n such that all the elements of $\Pi^{s}$ and $\Xi^{t}$ are positive for s≥m, t≥n. Let us define

(57) $$\Omega_{1}=\sum_{\tau =1}^{\bar{k}}\alpha^{\tau -1}{\left(\underset{\mathrm{length}=\;\tau -1}{\underbrace{F_{k-1}F_{k-2}\ldots F_{k-\tau +1}}}\right)}^{-T}\left(\sum_{j\in S}\sum_{r\in S}\pi_{i,j}^{\tau }\Xi_{j,\;r}^{\tau -1}{\left(H_{r,\;k-\tau +1}\right)}^{T}R_{r,k-\tau +1}^{-1}H_{r,\;k-\tau +1}\right){\left(F_{k-1}F_{k-2}\ldots F_{k-\tau +1}\right)}^{-1}$$

It should be noted that, under Assumption 5, $\Omega_{1}$ is definite positive for $\bar{k}\geq \max\left(m,n+1\right)$. Therefore, for $k\geq \bar{k}$, $Y_{i,k|k}^{}\geq \Omega_{1}>0$. Since $\bar{k}$ is finite, for $0\leq k\leq \bar{k}-1$, there exists a constant positive definite matrix $\Omega_{2}$ such that $Y_{i,k|k}^{}\geq \Omega_{2}>0$. Hence, there exists a positive definite matrix $\underline{\Omega }$ such that $0<\underline{\Omega }\leq Y_{i,\;k|k}^{}$. The proof is now complete. □

**Remark** **6.** *The result shown in Theorem 2 is only dependent on collective observability. This is distinct from some algorithms that require some sort of local observability or detectability condition [5,6,8,11,25], which poses a great challenge to the sensing abilities of sensors and restricts the scope of application.*

### 5.3. Convergence of Estimation Errors

In line with the boundedness of $Y_{i,k|k}^{}$ proven in Theorem 2, the convergence of local estimation errors obtained by the proposed DHIWCF is analyzed in this section. To facilitate the analysis, the following preliminary lemmas are required.

**Lemma** **3** [26,28,31]**.** *Given an integer N≥2, N positive definite matrices $M_{1},\ldots ,M_{N}$ and N vectors $v_{1},\ldots ,v_{N}$, the following inequality holds*

(58) $${\left(\sum_{i=1}^{N}M_{i}v_{i}\right)}^{⊤}{\left(\sum_{i=1}^{N}M_{i}\right)}^{-1}\left(\sum_{i=1}^{N}M_{i}v_{i}\right)\leq \sum_{i=1}^{N}v_{i}^{⊤}M_{i}v_{i}$$

**Lemma** **4** [26,28]**.** *Under Assumptions 4 and 5, and the proposed DHIWCF algorithm, if there exists a positive semidefinite matrix $\tilde{Y}$ such that $Y_{i,k|k}^{}\geq \tilde{Y},\;\forall k\geq 0,\;i\in S$, then there always exists a strictly positive scalar 0<β<1 such that*

(59) $$Y_{i,k+1|k}^{}\leq \beta {\left(F_{k}\right)}^{-T}Y_{i,\;k|k}^{}F_{k}^{-1}$$

For the sake of simplicity, let us denote the prediction and estimation error at node i by ${\tilde{x}}_{i,k|k-1}^{}={\hat{x}}_{i,k|k-1}^{}-x_{k}$ and ${\tilde{x}}_{i,k|k}^{}={\hat{x}}_{i,k|k}^{}-x_{k}$, respectively. The collective forms are, respectively, ${\tilde{x}}_{k|k-1}^{}=\mathrm{col}\left({\tilde{x}}_{1,k|k-1}^{},\ldots ,{\tilde{x}}_{N_{s},k|k-1}^{}\right)$ and ${\tilde{x}}_{k|k}^{}=\mathrm{col}\left({\tilde{x}}_{1,k|k}^{},\ldots ,{\tilde{x}}_{N_{s},k|k}^{}\right)$.

**Theorem** **3.** *Under Assumptions 4–6, the proposed DHIWCF algorithm yields an asymptotic estimate in each node of the network in that*

(60) $$\underset{k\to +\infty }{\lim}E\left\{{\hat{x}}_{i,k|k}-x_{k}\right\}=0,\;\forall i\in S$$

**Proof.**  Under Assumptions 4–6, Theorem 2 holds. Therefore, $Y_{i,k|k}^{}$ is uniformly lower and upper bounded. Let us define the following candidate Lyapunov function

(61) $$V_{i,k}(x)=x^{T}Y_{i,k|k-1}^{}x,\;i\in S$$

By virtue of Lemma 2, it can be concluded that there exists a positive real number $0<\tilde{\beta }<1$ such that

(62) $$Y_{i,k+1|k}^{}\leq \tilde{\beta }{\left(F_{k}\right)}^{-T}Y_{i,\;k|k}^{}F_{k}^{-1}$$

Then, one has

(63) $$\begin{array}{ll} V_{i,\;k+1}\left(E\left\{{\tilde{x}}_{i,k+1|k}^{}\right\}\right) & ={\left(E\left\{{\tilde{x}}_{i,k+1|k}^{}\right\}\right)}^{T}Y_{i,k+1|k}^{}E\left\{{\tilde{x}}_{i,k+1|k}^{}\right\}\\ & \leq \tilde{\beta }{\left(E\left\{{\tilde{x}}_{i,k+1|k}^{}\right\}\right)}^{T}{\left(F_{k}\right)}^{-T}Y_{i,\;k|k}^{}F_{k}^{-1}E\left\{{\tilde{x}}_{i,k+1|k}^{}\right\} \end{array}$$

Since $E\left\{{\tilde{x}}_{i,k+1|k}^{}\right\}=E\left\{F_{k}{\hat{x}}_{i,k|k}^{}-\left(F_{k}x_{k}+w_{k}\right)\right\}=F_{k}E\left\{{\tilde{x}}_{i,k|k}^{}\right\}$, one can obtain

(64) $$V_{i,\;k+1}\left(E\left\{{\tilde{x}}_{i,k+1|k}^{}\right\}\right)\leq \tilde{\beta }{\left(E\left\{{\tilde{x}}_{i,k|k}^{}\right\}\right)}^{T}Y_{i,\;k|k}^{}E\left\{{\tilde{x}}_{i,k|k}^{}\right\}$$

Notice that

(65) $$Y_{i,k|k}^{}=Y_{i,k|k}^{L}=\sum_{j\in S}\pi_{i,j}^{L}Y_{j,\;k|k}^{0}$$

(66) $${\hat{y}}_{i,k|k}^{}={\hat{y}}_{i,k|k}^{L}=\sum_{j\in S}\pi_{i,j}^{L}{\hat{y}}_{j,\;k|k}^{0}$$

Here, pre-multiplying (65) by $Y_{i,k|k}^{-1}$ and post-multiplying it by $x_{k}$ yields

(67) $$x_{k}=Y_{i,k|k}^{-1}\sum_{j\in S}\pi_{i,j}^{L}Y_{j,\;k|k}^{0}x_{k}$$

In a similar way, pre-multiplying (66) by $Y_{i,k|k}^{-1}$ yields

(68) $${\hat{x}}_{i,k|k}^{}=Y_{i,k|k}^{-1}\sum_{j\in S}\pi_{i,j}^{L}{\hat{y}}_{j,\;k|k}^{0}$$

Therefore,

(69) $$E\left\{{\tilde{x}}_{i,k|k}^{}\right\}=E\left\{{\hat{x}}_{i,k|k}^{}-x_{k}\right\}=Y_{i,k|k}^{-1}\sum_{j\in S}\pi_{i,j}^{L}E\left\{{\hat{y}}_{j,\;k|k}^{0}-Y_{j,\;k|k}^{0}x_{k}\right\}$$

According to (36), there is

(70) $${\hat{y}}_{j,\;k|k}^{0}-Y_{j,\;k|k}^{0}x_{k}={\left(1+d_{j}\right)}^{-1}\sum_{r\in J_{j}}Y_{r,k|k-1}^{}{\tilde{x}}_{r,k|k-1}^{}+\sum_{r\in J_{j}}{\left(H_{r,\;k}\right)}^{T}R_{r,k}^{-1}v_{r,\;k}$$

Since $E\left\{v_{r,\;k}\right\}=0$, one can get

(71) $$E\left\{{\tilde{x}}_{i,k|k}^{}\right\}=Y_{i,k|k}^{-1}\sum_{j\in S}\sum_{r\in J_{j}}\pi_{i,j}^{L}{\left(1+d_{j}\right)}^{-1}Y_{r,k|k-1}^{}E\left\{{\tilde{x}}_{r,k|k-1}^{}\right\}$$

Substituting (71) into (64) yields

(72) $$\begin{array}{l} V_{i,\;k+1}\left(E\left\{{\tilde{x}}_{i,k+1|k}^{}\right\}\right)\\ \leq \tilde{\beta }{\left[\sum_{j\in S}\sum_{r\in J_{j}}\pi_{i,j}^{L}{\left(1+d_{j}\right)}^{-1}Y_{r,k|k-1}^{}E\left\{{\tilde{x}}_{r,k|k-1}^{}\right\}\right]}^{T}Y_{i,\;k|k}^{-1}\left[\sum_{j\in S}\sum_{r\in J_{j}}\pi_{i,j}^{L}{\left(1+d_{j}\right)}^{-1}Y_{r,k|k-1}^{}E\left\{{\tilde{x}}_{r,k|k-1}^{}\right\}\right] \end{array}$$

Applying the fact that $Y_{i,\;k|k}^{}\geq \sum_{j\in S}\sum_{r\in J_{j}}\pi_{i,j}^{L}{\left(1+d_{j}\right)}^{-1}Y_{r,k|k-1}^{}$ and Lemma 3 to the right hand side of (72), one can obtain that

(73) $$\begin{array}{ll} V_{i,\;k+1}\left(E\left\{{\tilde{x}}_{i,k+1|k}^{}\right\}\right) & \leq \tilde{\beta }\sum_{j\in S}\sum_{r\in J_{j}}\pi_{i,j}^{L}{\left(1+d_{j}\right)}^{-1}{\left(E\left\{{\tilde{x}}_{r,k|k-1}^{}\right\}\right)}^{T}Y_{r,k|k-1}^{}E\left\{{\tilde{x}}_{r,k|k-1}^{}\right\}\\ & =\tilde{\beta }\sum_{j\in S}\sum_{r\in J_{j}}\pi_{i,j}^{L}{\left(1+d_{j}\right)}^{-1}V_{r,\;k}\left(E\left\{{\tilde{x}}_{r,k|k-1}^{}\right\}\right) \end{array}$$

Writing (73) for $i=1,2,\ldots ,N_{s}$ in a collective form, it turns out that

(74) $$V_{k+1}\left(E\left\{{\tilde{x}}_{k+1|k}^{}\right\}\right)\leq \tilde{\beta }\;\Pi^{L}\Xi \;V_{k}\left(E\left\{{\tilde{x}}_{k|k-1}^{}\right\}\right)$$

where

(75) $$V_{k}\left(E\left\{{\tilde{x}}_{k|k-1}^{}\right\}\right)=\mathrm{col}\left(V_{1,\;k}\left(E\left\{{\tilde{x}}_{i,k|k-1}^{}\right\}\right),\ldots ,V_{N_{s},\;k}\left(E\left\{{\tilde{x}}_{N_{s},k|k-1}^{}\right\}\right)\right)$$

and Ξ is a matrix with elements satisfying

(76) $$\Xi_{i,j}=\{\begin{array}{l} 1/\left(1+d_{i}\right),\;\mathrm{if}\;j\in J_{i}\\ 0,\;\mathrm{otherwise} \end{array}$$

Since the consensus matrix Π and the constructed matrix Ξ are both row stochastic, thus their spectral radiuses are both 1. As a consequence, for $0<\tilde{\beta }<1$, the elements of vector $V_{k+1}\left(E\left\{{\tilde{x}}_{k+1|k}^{}\right\}\right)$ vanishes as k tends to infinity in that $\underset{k\to +\infty }{\lim}E\left\{{\tilde{x}}_{k+1|k}^{}\right\}=0$. Due to the equation $E\left\{{\tilde{x}}_{k+1|k}^{}\right\}=F_{k}E\left\{{\tilde{x}}_{i,k|k}^{}\right\}$ and Assumption 4, it is straightforward to conclude that $\underset{k\to +\infty }{\lim}E\left\{{\hat{x}}_{i,k|k}-x_{k}\right\}=0$ for any i∈S. □

**Remark** **7.** *The Lyapunov function defined in (61) plays a crucial role in the convergence proof of the proposed algorithm, which can be easily extended to stability analysis of Kalman-like consensus filters in other scenarios. The reason for the non-singularity requirements of $F_{k}$ in Theorem 3 is that the proof of the Lyapunov method depends on Lemma 4, the establishment of which needs the invertibility of $F_{k}$.*

## 6. Experimental Results and Analysis

### 6.1. Simulation Setting

A target tracking scenario is adopted here to validate the effectiveness and superiority of the proposed DHIWCF. The centralized Kalman filter (CKF) is chosen as a benchmark to compare the proposed DHIWCF with following algorithms: The Kalman consensus filter (KCF), the generalized Kalman consensus filter (GKCF), the information weighted consensus filter (ICF), the consensus on information algorithm (CI), the consensus on measurements algorithm (CM), the hybrid consensus on measurement + consensus on information algorithm (HCMCI).

In the surveillance area, a target is moving with the discrete time linear model shown in (1). $x_{k}={\left[x_{k},y_{k},{\dot{x}}_{k},{\dot{y}}_{k}\right]}^{T}$ is the state vector at time instant k. $\left(x_{k},y_{k}\right)$ and $\left({\dot{x}}_{k},{\dot{y}}_{k}\right)$ are, respectively, the position and velocity components of the state. The state transition matrix $F_{k}$ and the process noise covariance matrix $Q_{k}$ are set as follows.

(77) $$F_{k}=\left[\begin{array}{cccc} 1 & 0 & 1 & 0\\ 0 & 1 & 0 & 1\\ 0 & 0 & 1 & 0\\ 0 & 0 & 0 & 1 \end{array}\right],\;Q_{k}=\left[\begin{array}{cccc} 10 & 0 & 0 & 0\\ 0 & 10 & 0 & 0\\ 0 & 0 & 1 & 0\\ 0 & 0 & 0 & 1 \end{array}\right]$$

The initial position of the target is randomly located at the 500×500 space. The initial speed is set to 2 units per time step, with a random direction uniformly chosen from 0 to 2π. In each simulation run, the initial prior error covariance is $P_{0}=\mathrm{diag}\left(100,100,10,10\right)$, and all nodes in the network share the same $P_{0}$. The initial prior estimate of each node is generated by adding zero-mean Gaussian noise with covariance $P_{0}$ to the true initial state. The total number of time steps is K=100 unless stated otherwise. The sampling time interval is T=1 s. 

The target of interest is observed by a number of networked sensors with measurement model shown in (2). The measurement matrix $H_{i,\;k}$ and the measurement noise covariance $R_{i,k}$ are given below.

(78) $$H_{i,\;k}=\left[\begin{array}{cccc} 1 & 0 & 0 & 0\\ 0 & 1 & 0 & 0 \end{array}\right],\;R_{i,k}=\left[\begin{array}{cc} 100 & 0\\ 0 & 100 \end{array}\right]$$

### 6.2. Performance Metrics

For a fair comparison, a total number $M_{c}=200$ of independent Monte Carlo runs are carried out. The consensus iterations L is set from 1 to 10. The consensus rate parameter is selected as $\varepsilon =0.65/d_{\max}$. For the proposed DHIWCF algorithm, the Metropolis weight matrix is chosen, which is computed only with knowledge of local node degree. Following metrics are chosen to evaluate the estimation performance from different aspects.

(1) The position root mean squared error (PRMSE), which indicates the tracking accuracy, is defined as

(79) $$\mathrm{PRMSE}=\sqrt{\frac{1}{M_{c}}\sum_{m=1}^{M_{c}}\frac{1}{N_{s}}\sum_{i=1}^{N_{s}}\left[{\left({\hat{x}}_{i,k}^{m}-x_{k}^{m}\right)}^{2}+{\left({\hat{y}}_{i,k}^{m}-y_{k}^{m}\right)}^{2}\right]}$$

where $\left({\hat{x}}_{i,k}^{m},\;{\hat{y}}_{i,k}^{m}\right)$ and $\left(x_{k}^{m},\;y_{k}^{m}\right)$ are, respectively, the estimated position and the true position in the m-th Monte Carlo run.

(2) The averaged position root mean squared error (APRMSE), which implies the overall tracking accuracy of an algorithm over all simulation runs, all time instants and all sensors, is defined as

(80) $$\mathrm{APRMSE}=\sqrt{\frac{1}{M_{c}}\sum_{m=1}^{M_{c}}\frac{1}{K}\sum_{k=1}^{K}\frac{1}{N_{s}}\sum_{i=1}^{N_{s}}\left[{\left({\hat{x}}_{i,k}^{m}-x_{k}^{m}\right)}^{2}+{\left({\hat{y}}_{i,k}^{m}-y_{k}^{m}\right)}^{2}\right]}$$

(3) The averaged consensus estimate error (ACEE), which indicates the degree of consensus among estimates from all nodes in the network, is defined as

(81) $$\mathrm{ACEE}=\frac{1}{M_{c}}\sum_{m=1}^{M_{c}}\frac{1}{N_{s}\left(N_{s}-1\right)}\sum_{i=1}^{N_{s}}\sum_{j=1}^{N_{s}}\sqrt{\left[{\left({\hat{x}}_{j,k}^{m}-{\hat{x}}_{i,k}^{m}\right)}^{2}+{\left({\hat{y}}_{j,k}^{m}-{\hat{y}}_{i,k}^{m}\right)}^{2}\right]}$$

(4) The normalized estimation error squared (NEES), which is used to check for filter consistency, is defined as

(82) $$\varepsilon_{k}={\left(x_{k}-{\hat{x}}_{k|k}\right)}^{T}P_{k|k}^{-1}\left(x_{k}-{\hat{x}}_{k|k}\right)$$

where $x_{k}$ and ${\hat{x}}_{k|k}$ are, respectively, the true state and estimated state. $P_{k|k}^{}$ is the posterior covariance at time instant k. Suppose that the filter is consistent, the NEES is subject to Chi-squared distribution with $n_{x}$ degrees of freedom. A way to check filter consistency is by testing the average NEES over a number of $M_{c}$ Monte Carlo runs, i.e.,

(83) $${\bar{\varepsilon }}_{k}=\frac{1}{M_{c}}\sum_{i=1}^{M_{c}}\varepsilon_{k}^{i}$$

Under similar assumptions $M_{c}{\bar{\varepsilon }}_{k}$ will be Chi-squared distributed with $M_{c}n_{x}$ degrees of freedom. Suppose the acceptance interval is $\left[r_{1},\;r_{2}\right]$, the Chi-square test is accepted if ${\bar{\varepsilon }}_{k}\in \left[r_{1},\;r_{2}\right]$. The filter is optimistic if the computed ${\bar{\varepsilon }}_{k}$ is much higher than $r_{2}$, while it is conservative with the computed ${\bar{\varepsilon }}_{k}$ below $r_{1}$. 

(5) Computational cost. The computational cost is defined as the averaged running time over all Monte Carlo runs.

### 6.3. Reuslts and Analysis

In this subsection, three simulation scenarios are chosen to evaluate and compare the estimation performance of the proposed DHIWCF algorithm with respect to the afore-mentioned metrics.

#### 6.3.1. Evaluation of the Effectiveness of the Proposed DHIWCF Algorithm

**Figure 2:**
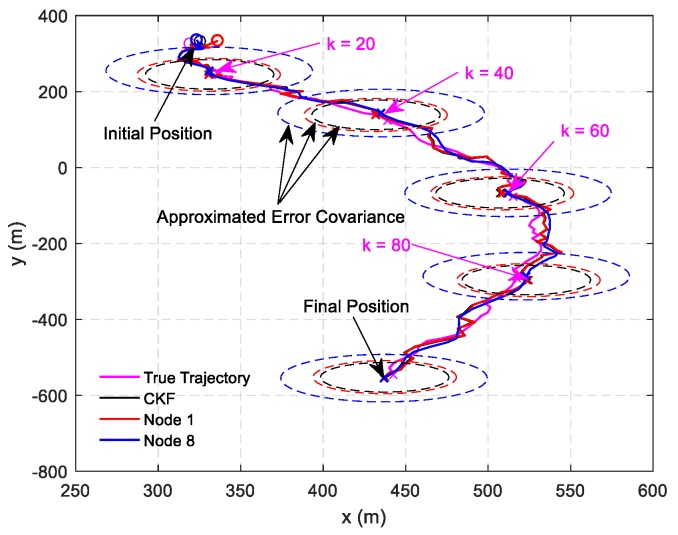
State estimation results: distributed hybrid information weighted consensus filter (DHIWCF) versus centralized Kalman consensus filter (CKF).

This scenario is designed to validate the effectiveness of the proposed DHIWCF algorithm. The target of interest is tracked by 8 networked sensors with a communication topology shown in Figure 1. Only node 1 can observe the target, then the node set {5,6,7,8} is naive about the target state. Figure 2 shows the estimated tracks obtained by local nodes with the proposed DHIWCF and the CKF. For simplicity, only the estimated tracks of node 1 and node 8 are plotted. To illustrate the evolution of each track, checkpoints are plotted in the same color every 20 steps. The covariance ellipses with 95% confidence at each checkpoint are plotted in dashed lines. As is shown, the true position (cross in black) is always enveloped by the corresponding ellipse in red (node 1) or blue (node 8), which validates the consistency of the local estimates. Compared with the CKF, the local estimates by the proposed DHIWCF is much more conservative. This is due to the fact that the network in Figure 1 has a weak connectivity and most nodes have poor joint observability. 

**Figure 3:**
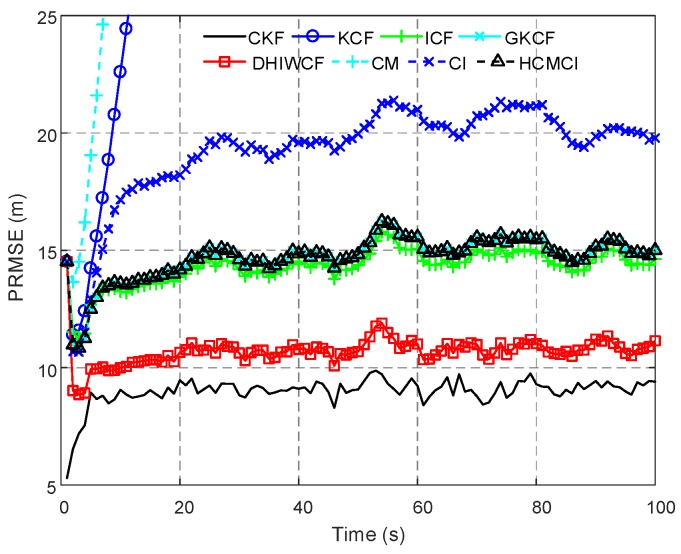
Position root mean squared error (PRMSE) averaged over all 8 nodes and 200 Monte Carlo runs.

In Figure 3, the PRMSE of the compared algorithms with a single consensus iteration is given. Both KCF and CM diverge in the considered scenario, while others can effectively track the target. Later on in this section KCF and CM are not considered for their poor performance. The proposed DHIWCF is more accurate for its lower PRMSE close to the CKF. Due to limited consensus iterations, GKCF, ICF, and HCMCI obtain PRMSE higher than DHIWCF, but is much lower than CI. 

**Figure 4:**
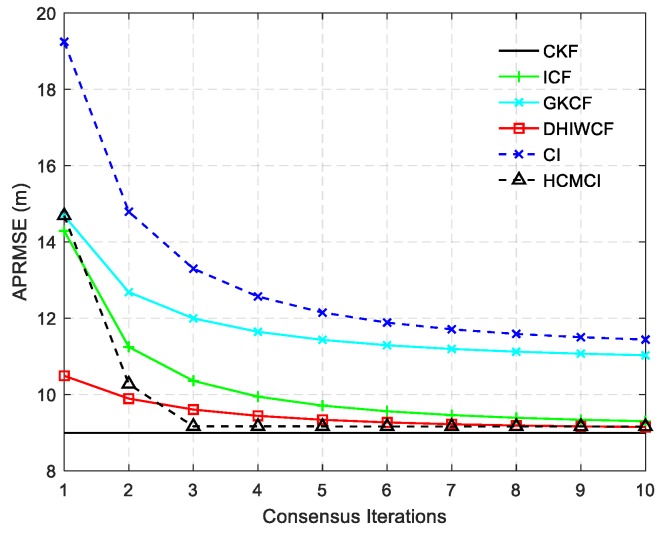
The averaged position root mean squared error (APRMSE) averaged over all nodes, all time steps and all Monte Carlo runs.

**Figure 5:**
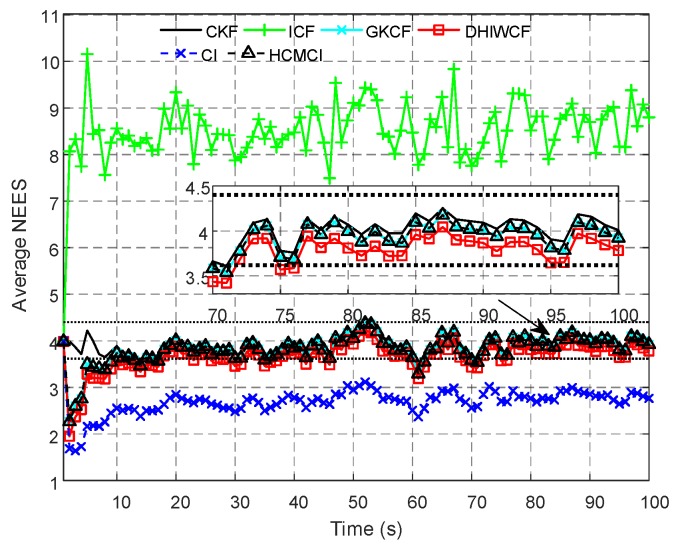
The average normalized estimation error squared (NEES) for the compared algorithms.

Figure 4 compares the APRMSE of different algorithms. It shows that GKCF and ICF obtain APRMSE much higher than that of ICF, HCMCI and DHIWCF. Specifically, DHIWCF performs the best with limited consensus iterations L≤2. As consensus iteration increases, DHIWCF asymptotically converges to the CKF. In addition, the performance of DHIWCF is a little much better than that of ICF. In Figure 5, the average NEES of different algorithms with a single consensus iteration is compared. It is obvious that the NEES curve of ICF lies much higher than the 95% concentration regions, which indicates that ICF has poor consistency in such a scenario. The NEES curve of CI always lies below the concentration regions, and hence its estimates is much conservative. The NEES curves of GKCF, HCMCI, DHIWCF, and CKF lie either below or within the concentration regions all the time steps, which shows an enhanced consistency.

**Figure 6:**
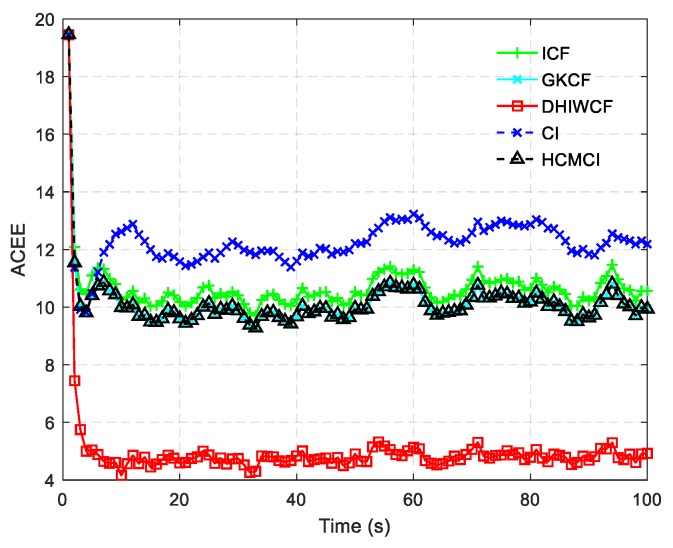
The averaged consensus estimate error (ACEE) for distributed algorithms.

**Figure 7:**
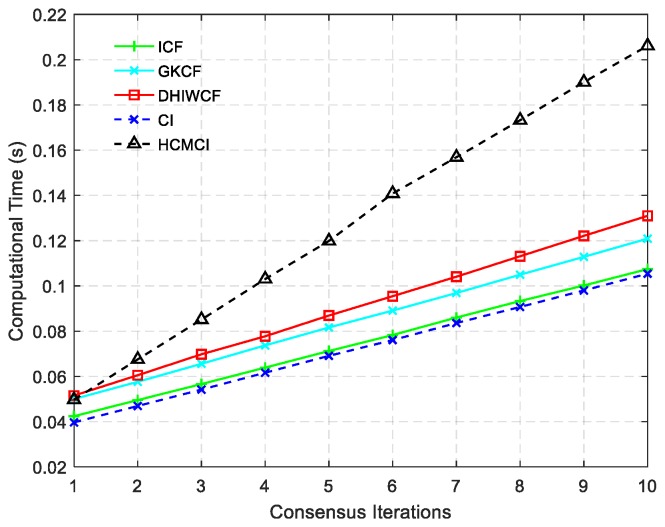
Computational cost of different algorithms.

The ACEE comparison of different algorithms with a single consensus iteration is shown in Figure 6. The proposed DHIWCF performs much better with regard to consensus in that it has relatively lower ACEE than other algorithms. Figure 7 shows the computational time with different number of consensus iterations. Although HCMCI performs a little better than DHIWCF in the aspect of APRMSE as shown in Figure 4, its ACEE and computational time are much higher than that of DHIWCF. Moreover, DHIWCF is a little more time-consuming than the most efficient CI as shown in Figure 7.

#### 6.3.2. Performance Comparison under Chain Topology

**Figure 8:**
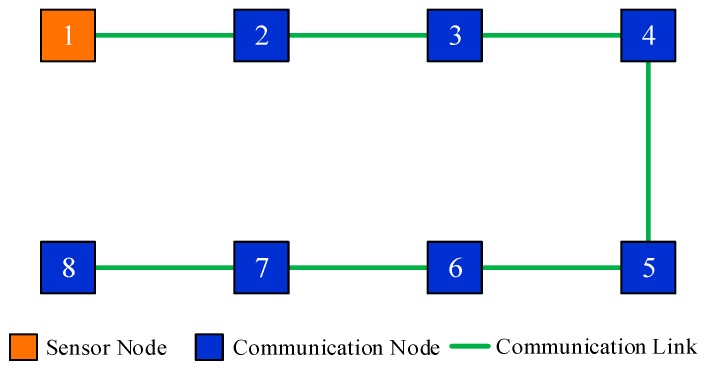
A sensor network with chain topology.

In this subsection, an even worse scenario is considered, where the networked sensors are connected with a chain topology as shown in Figure 8. As is illustrated, the target is observed only by node 1, and the remaining are communication nodes with no sensing abilities. Node set {3,4,5,6,7,8} and their immediate neighbors do not have measurement of the target, so they are naive about the target’s state information. It takes at least 7 consensus iterations for node 8 to be affected by node 1. As is shown in Figure 4, the APRMSE of GKCF and CI is much higher than that of ICF, HCMCI, and DHIWCF. Here, the estimation results of GKCF and CI are not considered.

**Figure 9:**
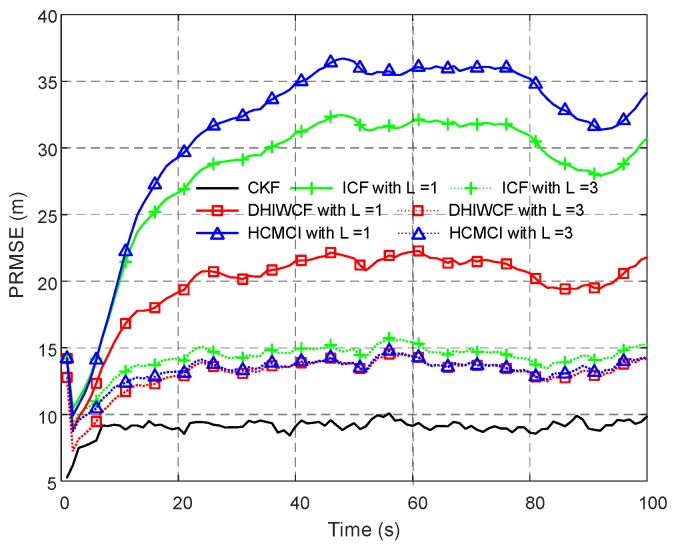
The averaged PRMSE for distributed algorithms with different consensus iterations.

**Figure 10:**
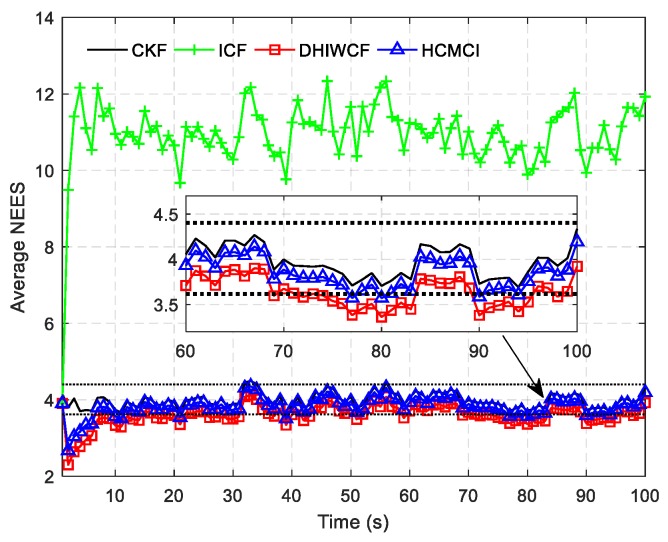
The averaged NEES of the compared algorithms.

In Figure 9, the PRMSE averaged over all nodes and all Monte Carlo runs for different consensus iterations is given. With a single consensus iteration, DHIWCF performs much better than ICF and HCMCI. When consensus iterations increase to L=3, DHIWCF is still smaller than the improved HCMCI. The result of averaged ACEE with a single consensus iteration is provided in Figure 10, which indicates that except for ICF, the remaining algorithms preserve good consistency. 

**Figure 11:**
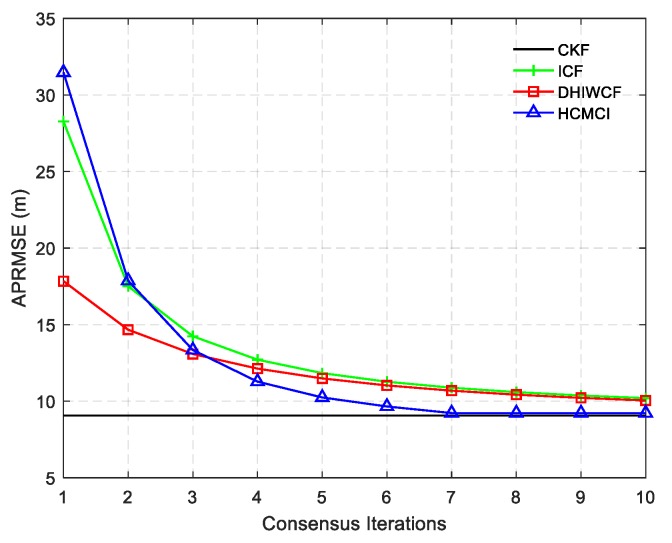
APRMSE for different algorithms under discussion.

**Figure 12:**
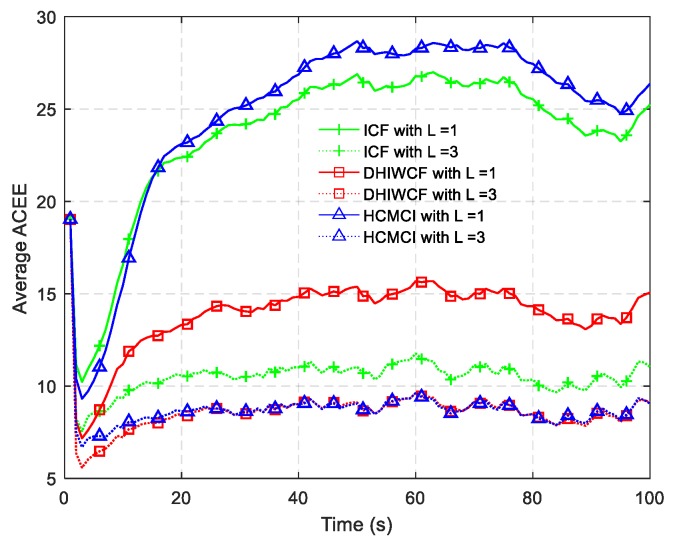
The average ACEE for distributed algorithms under discussion.

The APRMSE for the algorithms under discussion is plotted in Figure 11. Similar to the result in Figure 4, DHIWCF has smaller APRMSE with L≤3, and its performance approaches the KCF as consensus iterations progress. Although HCMCI obtains APRMSE a little smaller than DHIWCF, its average ACEE is relatively higher as shown in Figure 12, especially in the case L=1. Therefore, the proposed DHIWCF makes a good tradeoff between estimation accuracy and consensus on local estimates. 

#### 6.3.3. Performance Comparison in Large-Scale Sparse Sensor Networks

**Figure 13:**
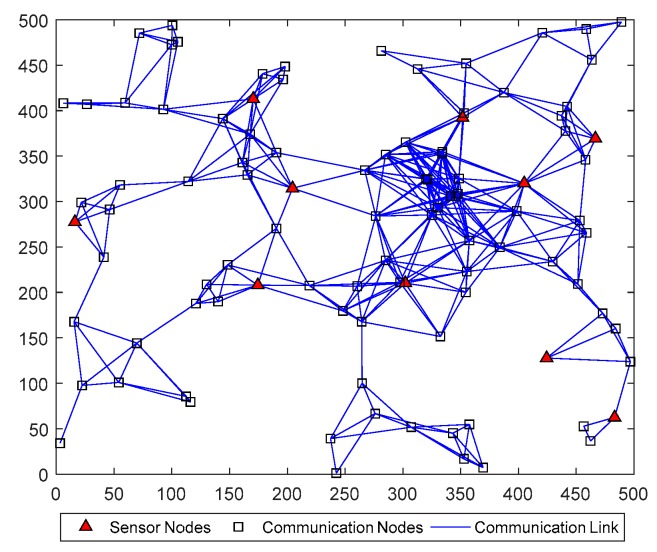
A large-scale sparse sensor network with 100 nodes.

This experiment is designed to test the performance of DHIWCF in large-scale sparse sensor networks. Assume that the interested target is tracked by 100 sensors, which are randomly located within the 500×500 space. The communication range of each sensor is set to be $R_{c}=10\sqrt{N_{s}}$. As is shown in Figure 13, there are 10 sensor nodes and 90 communication nodes in the surveillance area. The sensor nodes are able to observe the target, while the communication nodes act as relays of information among distant nodes and has no observation ability [24]. As is shown in Figure 13, most of nodes in the network are naive about the target’s state, which brings great challenges to target tracking.

**Figure 14:**
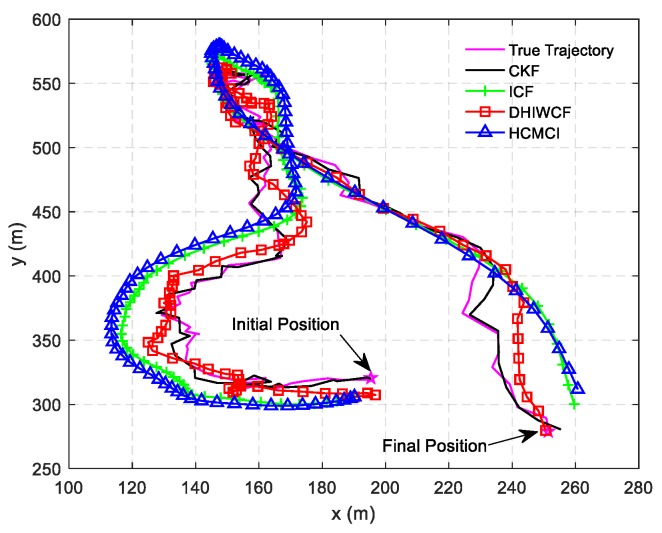
The true and estimated tracks of different algorithms.

**Figure 15:**
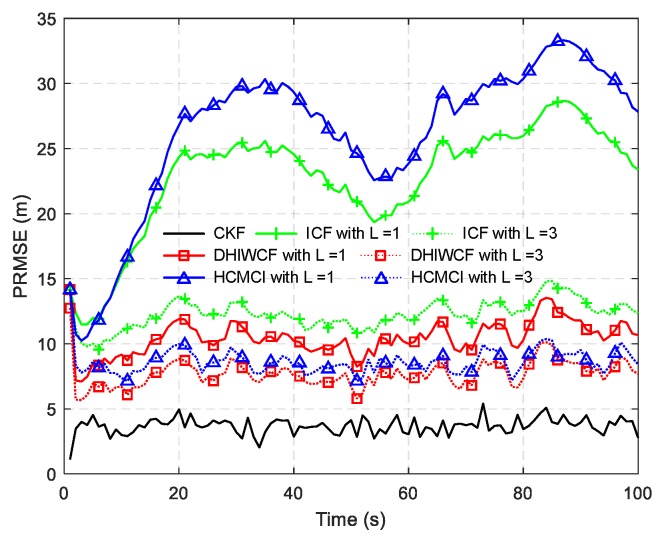
PRMSE for different algorithms in a spare sensor network.

**Figure 16:**
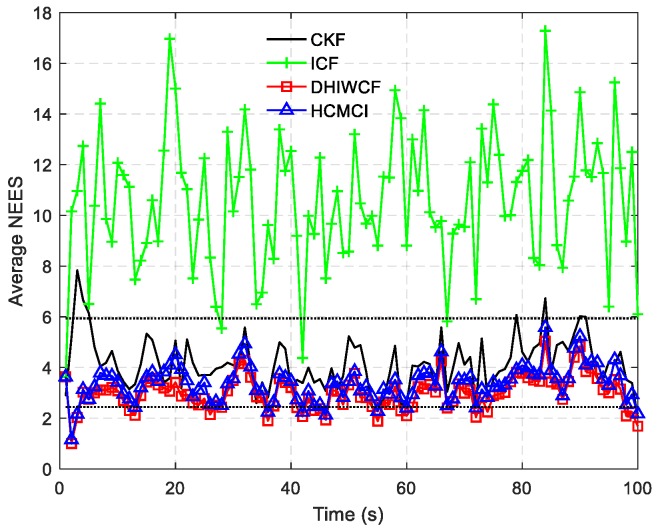
The averaged NEES for different algorithms in a spare sensor network.

In Figure 14, the estimated tracks by different algorithms with a single consensus iteration in a certain Monte Carlo run are plotted. It is intuitive to see that DHIWCF and KCF perform much better than ICF and HCMCI. Especially when the target suffers from relatively obvious process noise (for instance, the target suddenly changes its moving direction), DHIWCF recovers its estimate to the CKF more quickly. The results in Figure 15 further suggest that with limited consensus iterations, DHIWCF is able to obtain more accurate estimates than ICF and HCMCI in that it has relatively lower PRMSE compared with its counterparts. With respect to estimation consistency, it is shown in Figure 16 that the NEES curve of ICF lies higher than the concentration region, while the NEES curves of the remaining algorithms are always within or below the concentration region. Therefore, both HCMCI and DHIWCF show sound consistency on local estimates. 

To compare the overall performance of the distributed algorithms under discussion, Table 1 gives the APRMSE for different algorithms versus consensus iterations. Compared with ICF and HCMCI, the proposed DHIWCF has lower APRMSE. Especially in case of consensus iterations L≤3, the advantage is more obvious. This implies that DHIWCF is relatively more accurate. The computational time relative to that of CKF is investigated in Table 2, where RCT means the relative computation time. The proposed DHIWCF runs faster than HCMCI for fewer information exchanges. Although it takes less time for ICF to operate, the lower estimation accuracy and poor consistency make it not a good choice to estimate the state of interest. 

## 7. Conclusions

This paper considers the problem of distributed state estimation in presence of naive nodes with constrained communication resources. A novel distributed hybrid information weighted consensus filter, in which each node exploits not only the measurement information but also the prior estimate information from its immediate neighbors to update its local posterior estimate, is proposed. The proposed DHIWCF is able to settle the problem under consideration without any knowledge of global parameters, and preserve consistency of local estimates as well as achieve relatively high estimation accuracy and satisfactory consensus. Theoretical analysis with regard to consistency of local estimates, stability, and convergence of the estimator is also provided. The experimental results indicate that with limited consensus iterations, the proposed DHIWCF is much more accurate and reaches better consensus than the existing algorithms. In addition, DHIWCF preserves good consistency of local estimates in the experiments. Even a single consensus iteration is allowed, the proposed DHIWCF still performs much better. If more consensus iterations are available, the proposed DHIWCF would approach the performance of the centralized scheme. In the future research, a further investigation for distributed state estimation in mobile sensor networks, consensus protocol with event-triggered communication, more efficient design of consensus weights, and distributed nonlinear filtering problems and stability analysis will be taken into account.

## Figures and Tables

**Table 1:** Comparison of ARMSE for different algorithms.

APRMSE(m)	CKF	ICF	DHIWCF	HCMCI
1	3.68	22.87	10.44	26.24
2	3.68	14.80	9.23	11.93
3	3.68	12.28	8.37	8.64
4	3.68	11.05	7.63	7.93
5	3.68	10.30	7.46	7.78
10	3.68	8.56	6.87	7.25

**Table 2:** The relative computational time for different algorithms.

RCT	CKF	ICF	DHIWCF	HCMCI
L=1	1	5.26	7.92	9.49
L=2	1	6.58	10.84	15.55
L=3	1	7.30	13.71	21.57
L=4	1	8.34	16.68	27.04
L=5	1	9.39	19.55	32.25
L=10	1	14.18	34.43	60.05
